# Lignin Nanoparticles Deliver Novel Thymine Biomimetic Photo-Adducts with Antimelanoma Activity

**DOI:** 10.3390/ijms23020915

**Published:** 2022-01-14

**Authors:** Sofia Gabellone, Davide Piccinino, Silvia Filippi, Tiziana Castrignanò, Claudio Zippilli, Davide Del Buono, Raffaele Saladino

**Affiliations:** Department of Ecology and Biology, University of Tuscia, San Camillo De Lellis, 01100 Viterbo, Italy; sofia.gabellone@studenti.unitus.it (S.G.); d.piccinino@unitus.it (D.P.); silvia.filippi@unitus.it (S.F.); tiziana.castrignano@unitus.it (T.C.); zippillic@unitus.it (C.Z.); davide.delbuono@studenti.unitus.it (D.D.B.)

**Keywords:** lignin nanoparticles, drug delivery, pyrimidine photo-adducts biomimetic approach, antimelanoma activity

## Abstract

We report here the synthesis of novel thymine biomimetic photo-adducts bearing an alkane spacer between nucleobases and characterized by antimelanoma activity against two mutated cancer cell lines overexpressing human Topoisomerase 1 (TOP1), namely SKMEL28 and RPMI7951. Among them, Dewar Valence photo-adducts showed a selectivity index higher than the corresponding pyrimidine-(6-4)-pyrimidone and cyclobutane counterpart and were characterized by the highest affinity towards TOP1/DNA complex as evaluated by molecular docking analysis. The antimelanoma activity of novel photo-adducts was retained after loading into UV photo-protective lignin nanoparticles as stabilizing agent and efficient drug delivery system. Overall, these results support a combined antimelanoma and UV sunscreen strategy involving the use of photo-protective lignin nanoparticles for the controlled release of thymine dimers on the skin followed by their sacrificial transformation into photo-adducts and successive inhibition of melanoma and alert of cellular UV machinery repair pathways.

## 1. Introduction

Exposure to UV radiation represents a risk factor for photo-aging, epigenetic changes, suppression of the immune system, angiogenesis, nucleobase mutation, and emergence of melanoma [1]. UV radiation excites DNA pyrimidine bases, and in particular thymine [2], to corresponding singlet and triplet energy states yielding cyclobutane pyrimidine (CPD), pyrimidine-(6-4)-pyrimidone (6-4 PP), and Dewar Valence (DV) photo-adducts [3]. CPDs are three to four times more frequent in comparison with other photo-adducts [4]. UV-B is absorbed directly by DNA, while UV-A induces the formation of reactive radical species responsible for the oxidation of nucleobases and activation of pheomelanin electron transfer damage pathways [5,6]. To prevent genome mutations, the cell activates a complex network of repair pathways, including photo-damage recognition [7], cell cycle arrest [8], photo-adduct excision, and apoptosis [9,10]. The insurgence of melanoma can occur when these pathways are not effective [11]. In this context, the amount of photo-adducts produced during UV damage plays a key role [12]. The catabolic way of photo-adducts is still a matter of debate [9]. Recent reports suggest that they are released inside short oligonucleotides (30 mer) in a tight complex with the Transcription Factor II Human (TFIIH) and Replication Protein A (RPA) [9,13]. These oligonucleotides undergo a limited degree of degradation and can activate Mitogen-Activated Protein Kinase (MAPK) and checkpoint pathways involving tumor protein 53 (p53), Cyclin Dependent Kinases (CDK) inhibitor factor and p21, respectively, for the prevention of UV-damage, or in alternative, apoptosis [14]. In addition, they stabilize human Topoisomerase 1 (TOP1), affecting transcription bubble and replication fork [15]. Stable Topoisomerase Cleavage Complex (TOP1cc) forms near UV lesions preventing re-ligation processes [16,17]. In particular, TOP1 is trapped by oligonucleotide sequences containing photo-adducts with generation of Single-Strand Breaks (SSBs) [18]. Melanoma is one of the most aggressive forms of skin cancer, with limited therapeutic options. Since its incidence has been rapidly rising in recent years, the study of selective therapy has grown of interest [19]. The implication of nanoscience in the development of targeted therapies for melanoma shows multiple benefits and could significantly improve the outcome of melanoma patients [19,20]. Nanoparticles from natural sources are extensively investigated as drug delivery systems thanks to several benign factors, such as controlled release properties, good biocompatibility, biodegradability, and multifunctional and UV shielding properties [21]. May a combined strategy including the controlled release of thymine dimers and photo-adducts from nanoparticles be effective in the protection of the cell from UV damage and insurgence of melanoma?

## 2. Results

### 2.1. Summary and Concise Description of the Study

**Scheme 1:**
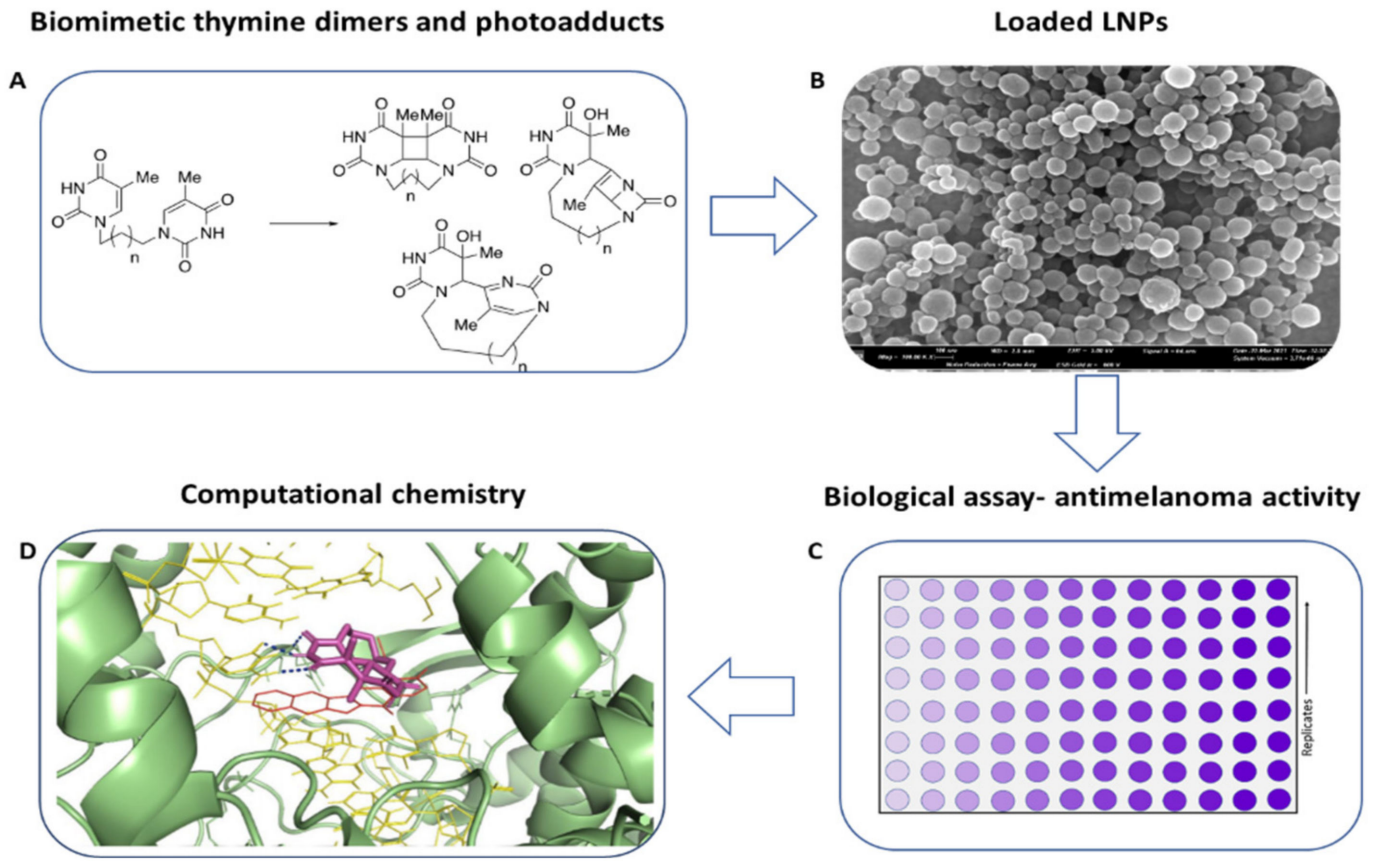
Experimental workflow. Panel (**A**): Synthesis of biomimetic compound and preparation of photo-adducts; panel (**B**): loading of biomimetic compound into LNPs and characterization. Panel (**C**): Biological assay of loaded-LNPs on melanoma cell lines. Panel (**D**): molecular docking of most active biomimetic compound.

We synthesized CPD, (6-4)PP, and DV photo-adducts from biomimetic thymine dimers characterized by a simple carbon side-chain spacer between the nucleobases. Pyrimidine dimers resembling thymidine residues have been previously reported as simple mimetics of DNA sequences [22,23], associated with (in a preliminary test) sunscreen protection against UV damage [24] (Scheme 1, panel A). Photo-adducts have been loaded inside lignin nanoparticles (LNPs) with high loading efficiency and capacity values and successively characterized for their stability and releasing properties, followed by the evaluation of anti-melanoma activity (Scheme 1, panel B). As a result, we observed an unprecedented antimelanoma activity of thymine photo-adducts against two mutated cell lines overexpressing TOP1, namely SKMEL-28 and RPMI7951. DV photo-adducts were more selective than (6-4)PP and CPD counterparts. The antimelanoma activity of these compounds was retained after loading into LNPs as a stabilizing and photo-protective drug delivery system (Scheme 1, panel C). In addition, docking computational studies showed the high stability of the interaction between DV photo-adducts and DNA/TOP1 complex (Scheme 1, panel D).

### 2.2. Synthesis of CPD, (6-4)PP, and DV Photo-Adducts by UV-Irradiation of Biomimetic Thymine Dimers ***4a-d***

**Scheme 2:**
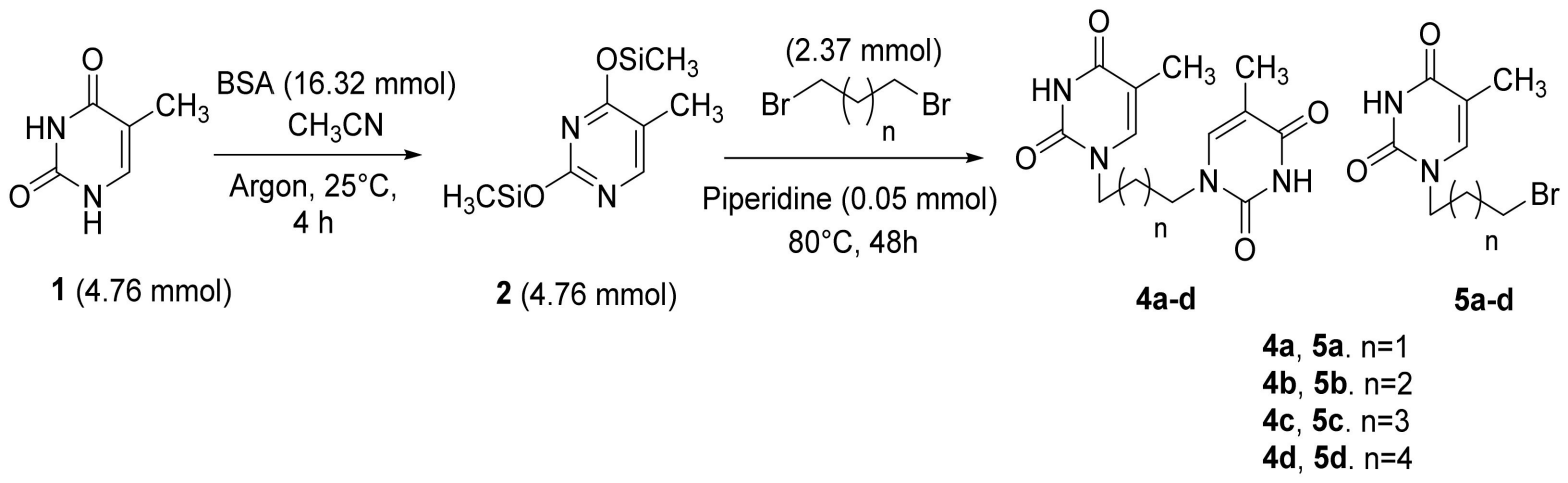
Synthesis of biomimetic thymine dimers **4a-d**.

We synthesized biomimetic thymine dimers **4a**-**d**, differing in the length of the spacer between the two nucleobases (from three to six carbon atoms), by a modification of the previously reported two-step procedure [22,23]. Briefly, thymine **1** (4.76 mmol) was treated with *N,O*-bis(trimethylsilyl)acetamide (BSA) in dry acetonitrile (CH_3_CN) at reflux temperature under argon atmosphere to afford *O*,*O*-bis-trimethylsilyl thymine **2** in quantitative yield (Scheme 2). Silylation procedures based on trimethylchlorosilane (TMCS)/trimethylamine (TEA) [22] and TMCS/hexamethyldisilazane (HMDS) [25] afforded **2** in lower yield and selectivity.

Tentative to alkylate **2** with 1,3-dibromo propane **3a** (2.37 mmol) in dry DMF (5.0 mL) at 170 °C [22] was unsuccessful in our hands, affording **4a** in very low yield, besides the unreacted substrate and mono-alkylated derivative **5a** (Table 1, entry 1). Better results were obtained performing the alkylation under bulk conditions. In this latter case, compound **2** was dissolved in 1,3-dibromo propane **3a** (2.37 mmol) in the presence of piperidine (5 μL) at 80 °C under argon atmosphere for 48 h. The dual role of piperidine in the neutralization of HBr (delivered during the reaction) and in removal the trimethylsilyl group is reported [26]. Under these experimental conditions, **4a** was obtained as the main reaction product in 85% yield, besides the low amount of **5a** (Scheme 2; Table 1, entry 2). The procedure was generalized for the alkylation of **2** with a panel of di-alkyl bromides including 1,4-dibromobutane **3b**, 1,5-dibromopentane **3c**, and 1,6-dibromohexane **3d**, to afford thymine dimers **4b**-**d** in high yield and quantitative conversion of substrate, besides the low amount of mono-alkylated derivatives **5b-d** (Scheme 2; Table 1, entries 3–5). Next, we evaluated the optimal conditions for the synthesis of photo-adducts, focusing on dimer **4a** as a representative selected case.

The photochemistry of thymine, simple *N*-methyl thymine, and thymidine derivatives has been reported [27,28,29,30,31,32,33] in different reaction solvents including water [27,28], acetonitrile [29,30,31], acetone, and mixtures [32,33]. Among them, acetone acts as a photosensitizer [34], favoring the synthesis of photo-adducts under lower-energy radiation doses [35,36,37].

**Scheme 3:**
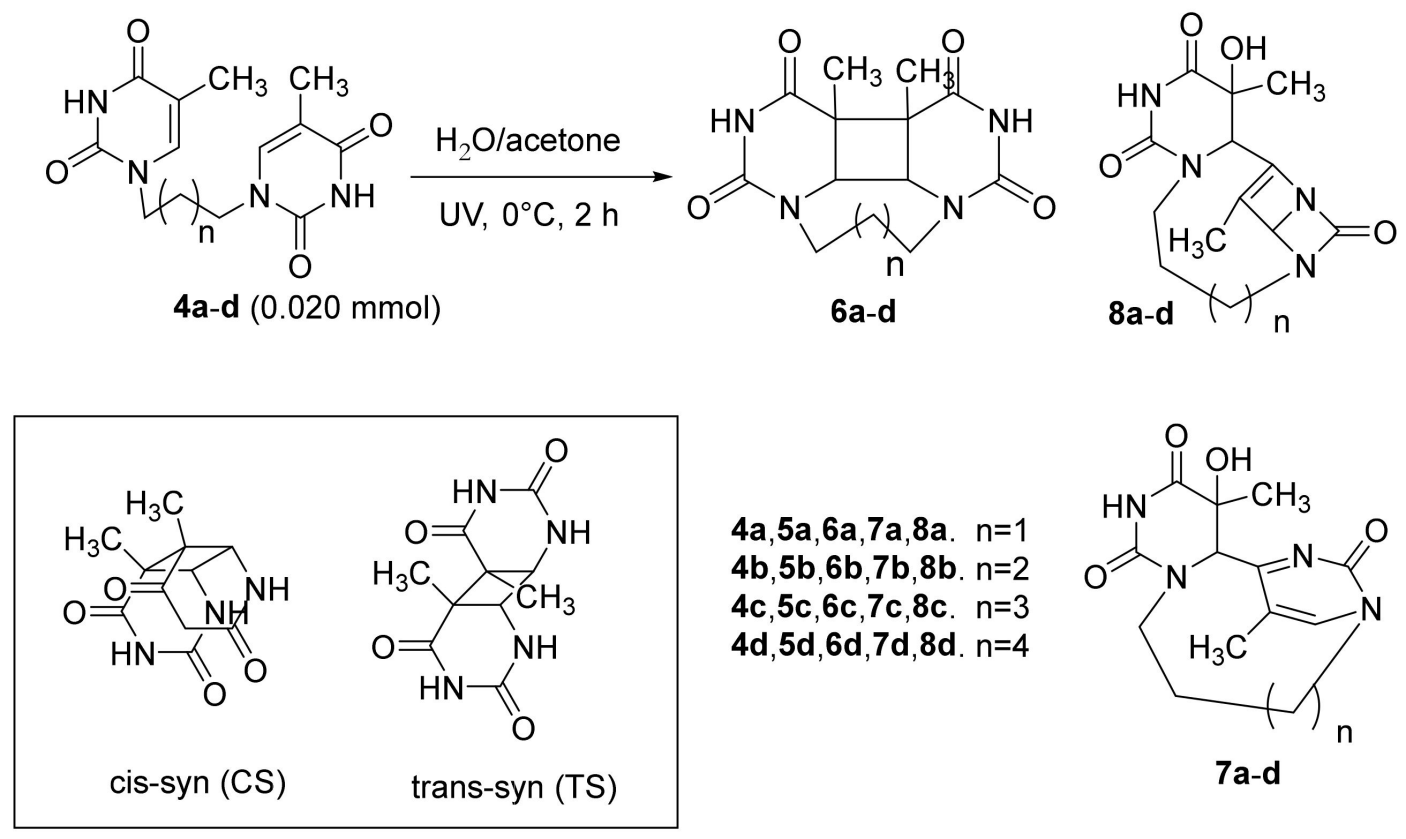
Synthesis of CPD, (6-4)PP, and DV photo-adducts by UV-irradiation of biomimetic thymine dimers **4a**-**d**.

We performed the photo-addition of **4a** in a Pyrex immersion well reactor (furnished of a refrigerator) with Haerus source 250 Watt (5.0 cm distance from the reactor; emission spectrum in Figure S1) in degassed deionized water (MilliQ)/acetone mixture (8:2 *v/v*, 100 mL) under argon atmosphere for 2.0 h at 25 °C and 0 °C. The irradiation of **4a** (0.17 mmol) at 25 °C quantitatively converted the substrate to afford CPD **6a**, (6-4)PP **7a**, and DV **8a** in low yield (Scheme 3; Table 2, entry 1) besides polar products not recovered under our experimental conditions. CPD **6a** was deprived of the adsorption band at 260 nm (C=C double bond), showing a λ_max_ value of 191 nm. This value was in accordance with data previously reported for CPD bearing 2′-deoxy-ribose/phosphate [38], or in alternative, a phosphoramidide-like backbone [39].

As revealed by the ^1^H-NMR analysis, **6a** was isolated as a mixture of trans-sin isomer (TS) (H-6 multiplet signals at 4.40 ppm and 4.27 ppm, respectively) and cis-sin isomer (CS) (H-6 singlet signal at 4.36 ppm) in 4.0:1.0 TS/CS ratio (Scheme 3, Table 2). The anti-isomer was not detected, probably due to steric effects of the spacer. Compounds **7a** and **8a** showed the expected ^1^H NMR signals at 7.48 ppm (H-6, singlet) and 5.32 ppm (H-6′, singlet), and 5.31 ppm (H-6, singlet) and 6.92 ppm (β-lactame bridge hydrogen, singlet), respectively. The mechanism of formation of (6-4)PP and DV photo-adducts is reported, focusing on the initial formation of an oxetane intermediate and successive intramolecular 4π electro-cyclization of the pyrimidone ring [40]. In order to improve the yield of photo-adducts, the reaction was repeated at 0 °C [27,28] to afford **6a**, **7a**, and **8a** in appreciable yield and quantitative conversion of substrate (Table 2, entry 2). Better results were finally obtained increasing the amount of acetone (water/acetone 6:4 *v*/*v*, 100 mL) at 0 °C, in which case photo-adducts were obtained in 76% total yield and quantitative conversion of substrate (Table 2, entry 3). These latter conditions were applied in the transformation of thymine dimer homologues **4b**-**d**, to afford CPDs **6b**-**d**, DVs **8b**-**d**, and (6-4)PPs **7b**-**c** from low to acceptable yield (Table 2, entries 4–6). Spectroscopical data of novel compounds were consistent with the expected structures, as well as full comparable with data previously reported for photo-adducts deriving from *N*-1 methyl thymine and thymidine derivatives [27,28,29,30,31,32,33,34,35,36,37]. Again, CPD-TS isomers prevailed with respect to the CS counterpart (Table 2, entries 4-6) [41,42,43]. As a general trend, the total yield of photo-adducts increased by increasing the carbon length of the spacer. In accordance with data reported for irradiation of DNA [44,45], CPDs were generally obtained as the major reaction products.

### 2.3. Lignin Nanoparticles as a Drug Delivery System for Biomimetic Thymine Dimers and Photo-Adducts

We evaluated the use of lignin nanoparticles (LNPs) as natural carriers for the drug delivery of biomimetic thymine dimers and photo-adducts [46]. LNPs meet the challenge of biocompatibility and photo-stability in drug-delivery systems, increasing the permeability across the skin barrier [47,48]. The capacity of LNPs to adsorb UV-A and UV-B is reported, and the role played by π-interactions between the aromatic sub-units of the polymer in the adsorption process has been deeply investigated [46,49]. In addition, LNPs are potent radical scavengers [21,46,49]. LNPs were prepared starting from Kraft Lignin (KL) by the nanoprecipitation technology [50] that requires the dissolution of the polymer in a primary solvent followed by addition of deionized water (secondary solvent) [51]. After the addition of water, lignin quickly aggregates in order to balance repulsive electrostatic forces, thus yielding stable colloidal nanoparticles that can entrap bioactive substances in their internal cavity [47,52].

**Figure 1:**
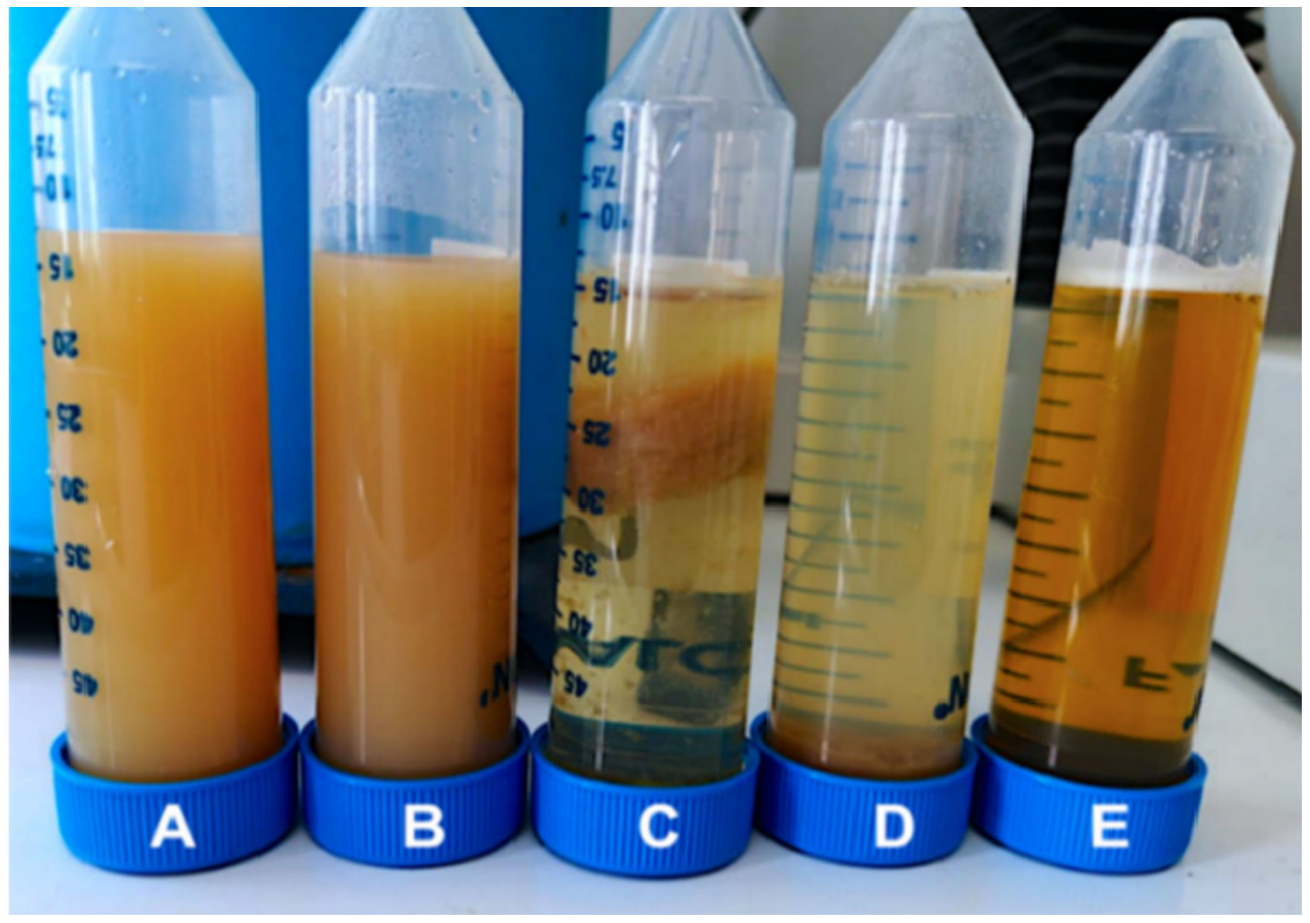
LNPs obtained during the nanoprecipitation process. (**A**) Stable colloidal LNPs (30 days at 25 °C). (**B**) Low stable colloidal LNPs (1–2 days at 25 °C). (**C**) Aggregates of LNPs. (**D**) Formation of a precipitate. (**E**) Absence of LNPs.

Initially, the optimal conditions to solubilize KL and **4a** in deionized water and dimethyl-isosorbide (DMI) were evaluated. DMI was selected as a primary solvent due to its green properties, being eco-certified by the European Chemical Agency (ECHA) [53]. In addition, DMI showed beneficial interactions with the epidermis, favoring the diffusion of bioactive compounds inside the corneous layer [54]. Compound **4a** (3.4 mM, 6.8 mM, 8.0 mM and 10 mM) and KL (1:5 *w*/*w*, 1:10 *w*/*w*, and 1:20 *w/w*, with respect to **4a**) were dissolved in DMI/water mixtures (1:1 *v*/*v*, 2:1 *v/v*, 3:1 *v*/*v*; total volume 3.0 mL) at 25 °C for 24 h. Irrespective from the concentration of **4a** and KL, the optimal DMI/water ratio was 2:1 *v*/*v*. Nanoprecipitation was then performed by adding different amounts of deionized water (from 3.0 mL to 24 mL, respectively). Results are reported in Table 3 and Figure 1 and depended on the amount of water we obtained: (i) stable colloidal LNPs (30 days at 25 °C; Figure 1A); (ii) low stable colloidal LNPs (1–2 days at 25 °C; Figure 1B); (iii) aggregates of LNPs (Figure 1C); (iv) formation of precipitate (Figure 1D); and (v) absence of LNPs (Figure 1E).

In particular, the addition of 3.0 mL of deionized water always produced a precipitate (Table 3, entry 1), while stable colloidal LNPs were obtained (1:5 *w/w* and 1:10 *w/w* **4a**/KL ratio, respectively) using both 6.0 mL and 12 mL of deionized water, respectively (Table 3, entry 2 and entry 3).

A further increase in the secondary solvent afforded low stable colloidal LNPs (18 mL; Table 3, entry 4) and aggregates (24 mL; Table 3, entry 5). Finally, LNPs were not formed after the addition of high volumes of deionized water (Table 3, entries 6–8). The optimal conditions affording stable colloidal LNPs (1:5 *w*/*w* and 1:10 *w*/*w* **4a**/KL ratio, 3.0 mL of DMI/water 2:1 *v*/*v*, and 6.0 mL of secondary solvent) were successively applied for the encapsulation of **4b**-**d**, and of the most abundant photo-adducts **6a**-**d** and **8a-d**. The loading capacity (LC) and loading efficiency (LE) Equations (1) and (2) were measured as a function of the concentration of the encapsulated compound [55,56,57,58].

LNPs were separated by centrifugation, and the supernatant was analyzed by HPLC at λ_max_ value of the encapsulated compound (271 nm for **4a**-**d**, 191 nm for **6a**-**d**, and 192 nm for **8a**-**d**, respectively). LC and LE are reported in Table 4. Irrespective from the experimental conditions, the highest LE and LC were obtained in the presence of the highest value of the compound/KL ratio (compound/KL ratio of 1:5) [59]. These results are of the same order of magnitude than that previously reported for the encapsulation of other bioactive natural substances inside LNPs [60].

(1) $$\mathrm{LC}\;\left(\%\right):\frac{\left(weight\;of\;starting\;drug-weight\;of\;unloaded\;drug\right)}{weight\;of\;lignin}$$

(2) $$\mathrm{LE}\;\left(\%\right):\frac{\left(weight\;of\;starting\;drug-weight\;of\;unloaded\;drug\right)}{weight\;of\;starting\;drug}$$

As a general trend, **4a**-**d** generally showed LE and LC values higher than that of the corresponding photo-adducts **6a**-**d** and **8a**-**d** (Table 4, entries 1–4 versus entries 5–12). In addition, LE and LC increased by increasing the number of carbon atoms in the spacer (see for example Table 4, entry 4 versus entries 1–3), highlighting the role played by hydrophobicity in the nucleation of lignin and bioactive compounds during the loading process [61,62]. The role of hydrophobicity in the encapsulation of drugs inside lignin nanoparticles is well reported, and the role played by Van der Walls, hydrogen bonding, and π–π stacking interactions in this process have been deeply investigated [63,64].

**Figure 2:**
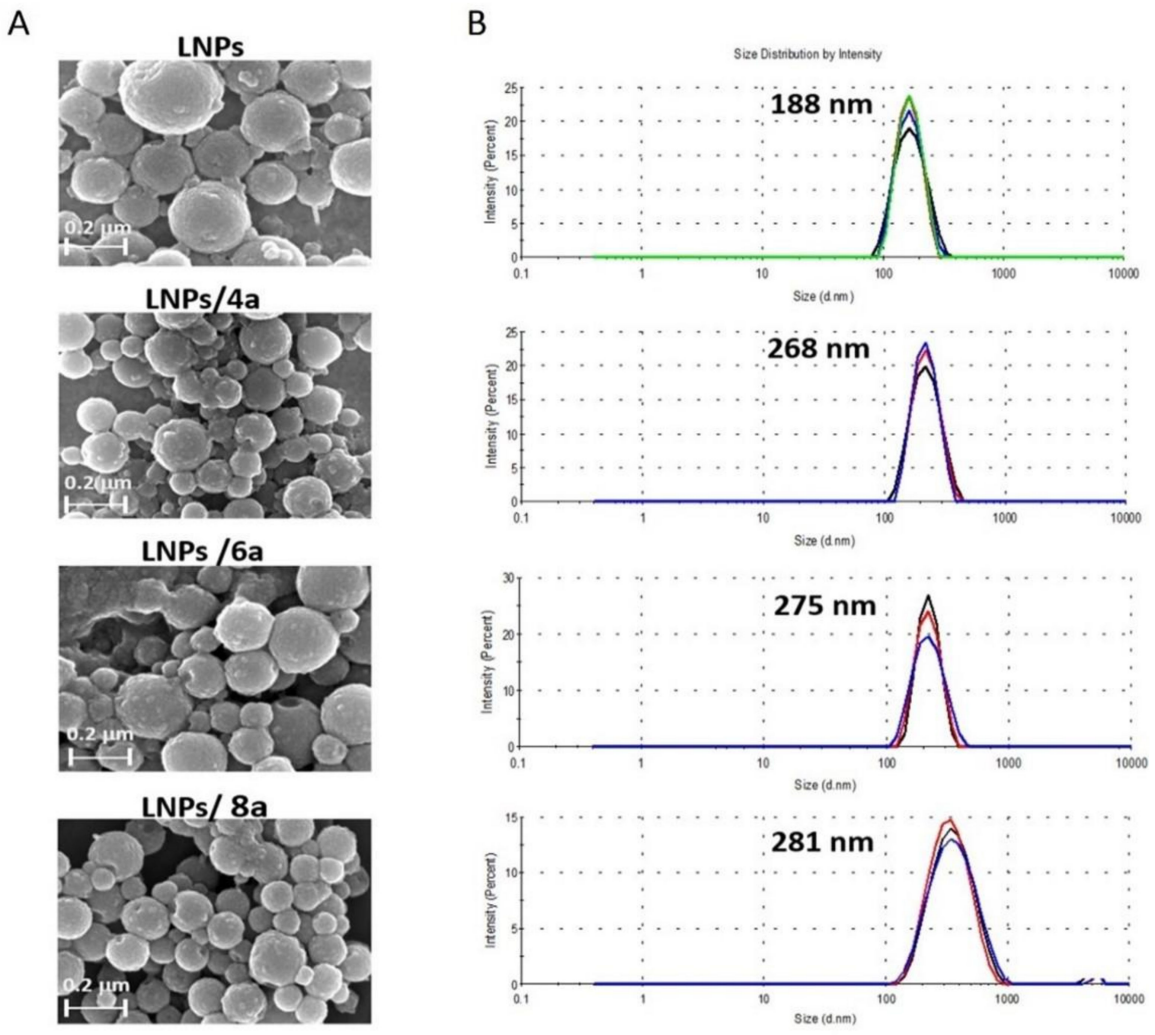
Morphological analysis of loaded and unloaded nanoparticles. Panel (**A**): Scanning electron microscopy of empty LNPs, and loaded LNPs/**4a**, LNPs/**6a**, and LNPs/**8a**. Panel (**B**): Dynamic light scattering analysis.

Loaded and unloaded LNPs showed a regular spherical shape with the surface characterized by a rough aggregate of small clumps (Figure 2, Panel A). Crystals were not present on the surface of loaded particles [65]. In addition, some LNPs/**6a** and LNPs/**8a** showed holes in their structure. The presence of these holes may be due to jet collision-like processes involving nanoclusters of lignin and DMI [66] and successive rush-out effects of the solvent during drying [67]. Dynamic light scattering analysis showed monodisperse nanoparticles. Different values of the average diameter were found depending on the presence or absence of loaded compounds. Empty LNPs showed an average particle diameter of 188 nm lower than LNPs/**4a** (268 nm), LNPs/**6a** (275 nm), and LNPs/**8a** (281 nm), respectively (Figure 2, Panel B). In addition, the diameter of LNPs/**6a** and LNPs/**8a** was larger than that of the thymine dimer counterpart (compound **4a**), in accordance with the expected higher steric hindrance of photo-adducts.

### 2.4. UV Shielding Capacity of Loaded LNPs

**Figure 3:**
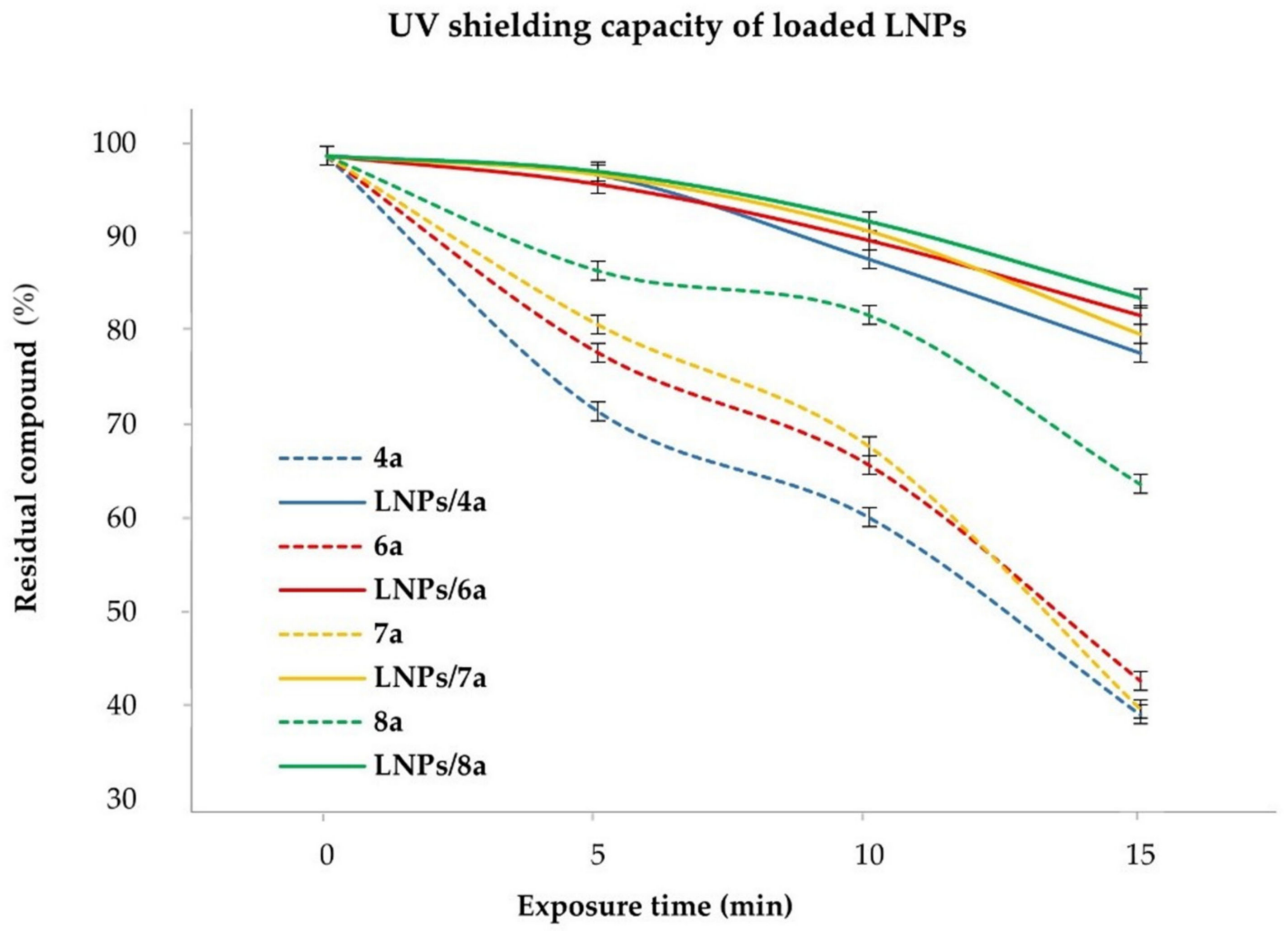
UV shielding capacity of LNPs. The photo-protective effect is expressed as the amount of residual **4a**, **6a**, **7a**, and **8a** recovered after UV treatment of LNPs/**4a**, LNPs/**6a**, LNPs/**7a**, and LNPs/**8a**.

The UV shielding capacity of LNPs allows them to protect bioactive compounds from premature deactivation [21]. To evaluate the photo-protective role of LNPs we studied the irradiation of LNPs/**4a**, LNPs/**6a**, LNPs/**7a**, and LNPs/**8a** as a selected representative example of thymine dimers. The samples were suspended in DMI/water mixture (2:1 *v*/*v*, 1.0 mL) and treated with UV under previously reported experimental conditions. At scheduled times (5, 10, and 15 min), aliquots (0.2 mL) of the suspension were withdrawn and analyzed. An excess of DMI (1.0 mL) was added to disaggregate LNPs, followed by filtration of residual lignin. The residual concentrations of **4a**, **6a**, **7a**, and **8a** (expressed as % with respect to the starting material) were evaluated at λ_max_ 271 nm. Data were compared with **4a**, **6a**, **7a**, and **8a** after irradiation under similar experimental conditions (Figure 3). Irrespective from the experimental conditions, LNPs showed an excellent photo-protective capacity after 15 min of irradiation, reaching in the case of compound **7a** a photo-protection capacity of almost 40%.

### 2.5. Releasing Properties

**Figure 4:**
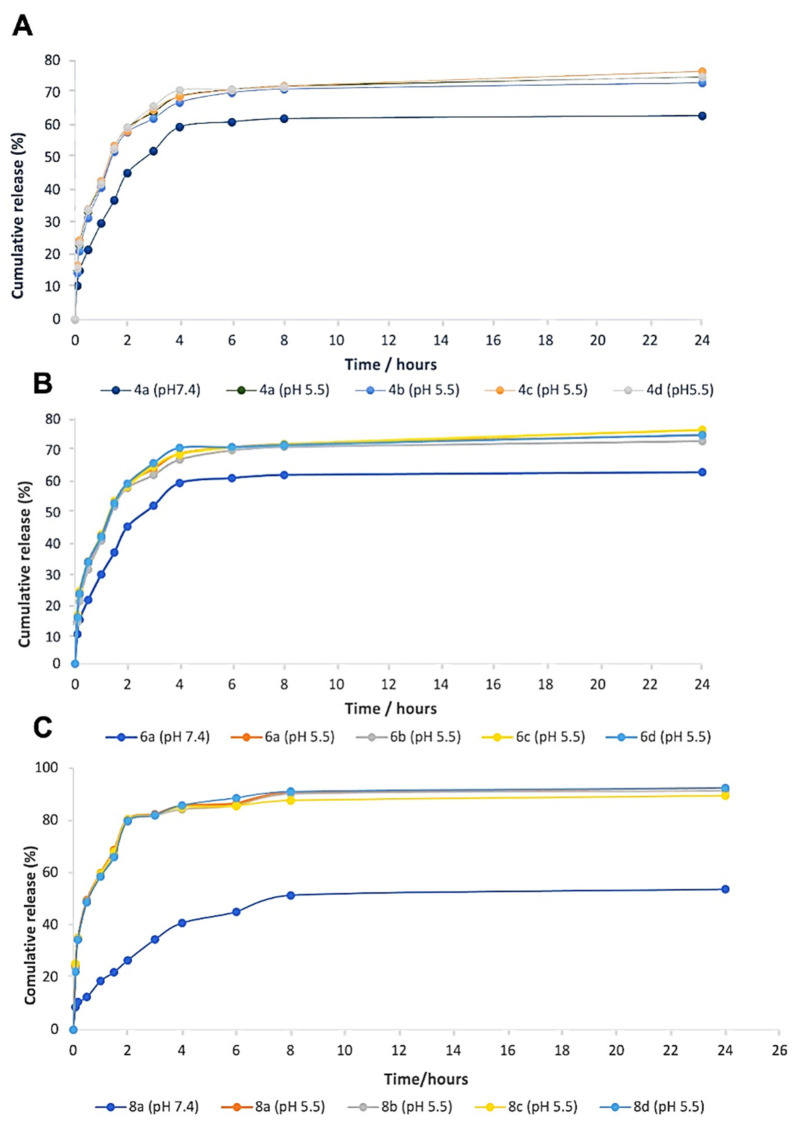
The cumulative releasing property of biomimetic thymine dimers **4a**-**d** (**A**) and photo-adducts **6a**-**d** (**B**) and **8a**-**d** (**C**).

The cumulative releasing capacity of LNPs was evaluated as previously reported in the literature [68] and normalized with respect to the total amount of entrapped compound. In a typical procedure, the appropriate LNPs (7.5 mg) were suspended in water (3.0 mL) and dialyzed against deionized water (60.0 mL) at two selected pH values (pH 7.4 and 5.5, respectively) at 37.5 °C for 24 h [69]. The amount of released compound was measured by UV–vis spectrophotometry at the optimal λ_max_ value for each compound by sampling the dialysis solution at appropriate time intervals (Figure 4). The withdrawn volume was replaced by corresponding deionized water in order to maintain constant the volume of the solution (Section 3).

Firstly, we studied the kinetic release of **4a** at λ_max_ 271 nm. LNPs/**4a** exhibited a fast release of **4a** at both pH values during the first 4 h (57% at pH 7.4, and 68% at pH 5.5, respectively), reaching a plateau within 24 h (60% at pH 7.4, and 70% at pH 5.5, respectively) (Figure 4, Panel A). The overall efficacy of the process was favored at pH 5.5 in accordance with the effect exerted by acidic conditions in the opening of the pores of LNPs [70]. The cumulative releasing capacity of **4b**-**d** was studied at the optimal pH value of 5.5. LNPs/**4b**-**d** showed a kinetic release similar to **4a**, compound **4d** being released in the highest amount (71% within 4 h) (Figure 4, Panel A). The cumulative releasing effect of LNPs/**6a**-**d** (Figure 4, Panel B) and LNPs/**8a**-**d** (Figure 4, Panel C) at pH 5.5 (measured at optimal λ_max_ 191 nm, and λ_max_ 192 nm, respectively) was similar to that of LNPs/**4a**-**d** and higher than LNPs/**8a**-**d.**

### 2.6. Biological Activity

Preliminary sunscreen data of **4a**-**d** have been reported focusing on SKH1-E cell line, human abdominal skin explants, and in vivo hairless mouse models [23]. Data for photo-adducts **6a**-**d**, **7a**-**d**, and **8a**-**d** are not available. Thymine photo-adducts are well recognized as responsible for the activation of different molecular pathways involved in the cellular response to UV damage [71], including DNA repair system, melanogenesis [72,73], and Nucleotide Excision Repair (NER) system [74,75,76,77,78]. In addition, TOP1ccs trapped near photo-adducts activates BER and remove (6-4)PPs in NER-deficient cells [4]. TOP1 is highly expressed in malignant tumors, including carcinomas of the colon, prostate, ovary, lung, and melanoma. These data associated with the active role played by photo-adducts in melanogenesis [79] open the possibility for these compounds to possess antimelanoma activity. For this reason, we decided to study the antimelanoma activity of **4a**-**d**, **6a**-**d**, and **8a**-**d** against two melanoma cancer cell lines, namely SK-MEL28 and RPMI7951 bearing a different genetic background [80]. The antimelanoma activity was determined using the MTT cell viability assay, following the absorbance change (λ_max_ 570 nm and 630 nm). The human FB789 cell line was used as reference. The CC_50_ (cytotoxic concentration that causes death of 50% of viable cells), IC_50_ (minimum concentration inhibiting 50% of the melanoma cells), and Selective Index (SI) (ratio between IC_50_ of reference cell line versus melanoma cell line) values are reported in Table 5. Compounds were not toxic after 24 h treatment in the FB789 cell line (IC_50_ > 100 μg/mL; Table 5, entries 1–11), with the only exception of **8d** (Table 5, entry 12). In the case of **4a**-**d** this result was in accordance with data previously reported [23]. Compounds **4a**-**d** were ineffective against melanoma cell lines. Instead, photo-adducts showed appreciable antimelanoma activity and selectivity (Table 5, entries 1-4). In the case of the CPDs family, **6b** showed the highest SI value (Table 5, entry 6 versus entries 5 and 7-8). DVs **8b** and **8d** showed the highest activity against SK-Mel 28 (Table 5, entries 10 and 12, respectively), while **8a** and **8c** were effective against RPMI7951 (Table 5, entries 9 and 11, respectively). In addition, high values of SI were obtained for **8a** and **8b**, compound **8b** being the most selective one with SI value of c.a. 234. Next, the antimelanoma activity of compounds **8a** and **8b** was evaluated after loading inside LNPs.

The kinetic release of LNPs/**8b** in the culture medium (Dulbecco’s modified eagle medium) is reported in Figure S2 as a selected sample and compared with data referring to the buffer medium at pH 5.5 (Section 2.4). Compound **8b** was released faster in DMEM than in buffer, accordingly to data previously reported [81]. LNPs alone showed no toxicity against the FB789 cell line (Table S1). In this latter case, the antimelanoma activity was measured at three different times (2, 4, and 24 h) in order to evaluate the effect of the kinetic release of the compound on the biological activity. LNPs/**8a** and LNPs/**8b** retained the antimelanoma activity against both SK-Mel 28 and RPMI7951 cell lines, respectively (Table 5, entries 13 and 14). Note that the antimelanoma activity of LNPs/**8a** and LNPs/**8b** increased during the time. In addition, LNPs/**8a** and LNPs/**8b** showed values of SI of the same order of magnitude than **8a** and **8b**, confirming the efficacy of LNPs in the drug delivery process. Note that the recorded SI values were higher than those, or of the same order of magnitude as, the approved drugs such as camptotechin, cisplatin, and doxorubicin [82,83].

### 2.7. In Silico Molecular Docking Analysis

In silico molecular docking analysis was performed to evaluate binding affinity, binding conformation, and non-covalent interactions between DVs **8a** and **8b** and TOP1 in complex with 22 base pairs DNA duplex (PDB ID: 1T8I) [84]. Compounds **4a** and **4b** were used as references. The active site of the TOP1/DNA duplex was characterized by amino acids Arg364 and Asp533, and nucleotides DC112, DA113, and DT10, as the most relevant residues [85]. In order to obtain well-balanced and reliable intermolecular interactions, we performed an energy minimization run of the system using the Amber99 force field [84] provided by Gromacs 2020.3 (www.gromacs.org).

DVs **8a** and **8b** showed higher TOP1/DNA duplex binding affinity than corresponding dimers **4a** and **4b** (Table 6, entries 1, 2 versus entries 3, 4), **8b** having the highest binding affinity (Table 6, entry 2). These data are in accordance with the trend of the antimelanoma activity previously reported. The conformations of ligands with the best binding affinity towards the TOP1/DNA duplex are in Figure S3.

**Figure 5:**
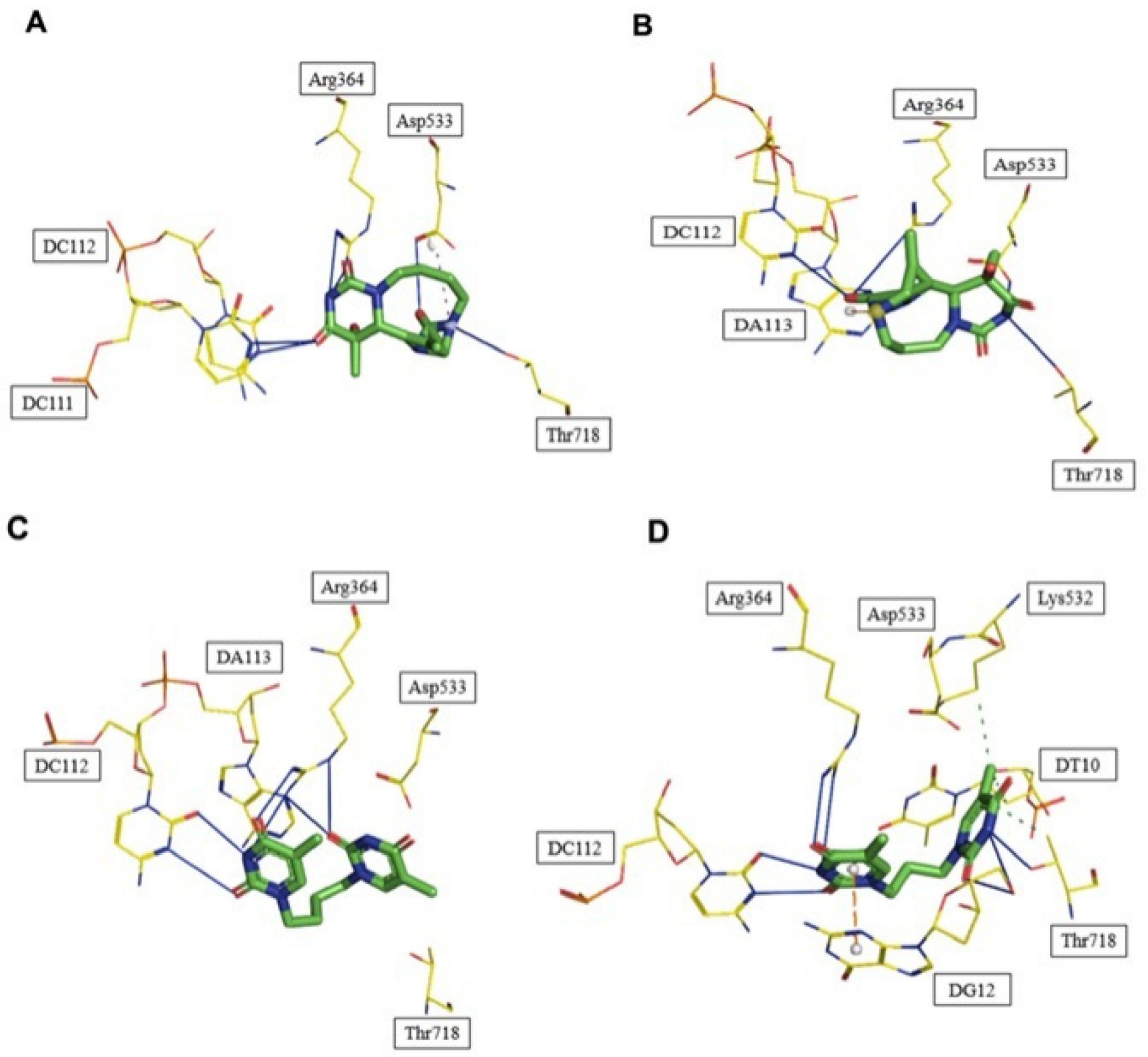
Panel (**A**): Binding mode of compound **8b**. H-bond interactions are shown with continuous blue lines, and salt bridges are reported as dashed magenta lines. Panel (**B**): Binding mode of compound **8a**. H-bond interactions are shown with continuous blue lines, and pi–cation interactions are reported as dashed red lines. Panel (**C**): Binding mode of compound **4b**. H-bond interactions are shown with continuous blue lines. Panel (**D**): Binding mode of compound **4a**. H-bond interactions are shown with continuous blue lines, hydrophobic interactions with dashed green lines, and pi-stacking interactions with dashed orange lines.

The non-covalent interactions between compounds **8a**-**b** and **4a**-**b**, and TOP1/DNA duplex, were calculated through the protein–ligand interaction profiler PLIP 2021 [86], by using AutoDock Vina for input poses. Compound **8b** (Table 6, entry 2) showed one H-bonding interaction with DC111, DC112, Asp533, and Thr718 and two H-bonding interactions with Arg364. This compound also formed a salt bridge with Asp533 (Figure 5, Panel A). Similar H-bonding interactions were observed for **8a** (Table 6, entry 1). In this latter case, only one H-bonding interaction was observed with Arg364, and no salt interaction was operative with Asp533 (Figure 5, Panel B). The higher number of non-covalent interactions of **8b** with Arg364 and Asp533 may be responsible for the highest antimelanoma observed for this compound. This hypothesis is in accordance with the AutoDock Vina’s binding affinity rank. Regarding thymine dimers, **4b** (Table 6, entry 4) formed H-bonding interactions with DC112, DA113, and Arg364. Instead, **4a** (Table 6, entry 3) interacted with DG12, DC112, Arg364, and Thr718. Hydrophobic interactions were observed only for **4a**. Note that neither of the two dimers **4a** and **4b** (Table 6, entries 3 and 4) formed non-covalent interactions with Asp533, suggesting a lower efficacy of dimers in the interaction with TOP1/DNA duplex with respect to **8a**-**b** (Figure 5, Panels C and D, respectively).

## 3. Materials and Methods

Kraft lignin was purchased from Sigma Aldrich (St. Louis, MO, USA) and purified before use by a standard procedure, including alkali-acid treatment and continuous washing with deionized water. 1,3-Dibromopropane, 1,4-dibromobutane, 1-5-dibromopentane, 1-6-dibromohexane, *N,O*-bis-(trimethylsilyl)-acetamide, silica gel (high-purity grade, 230–400 mesh particle size), dialysis bag (MWCO: 1 kDa), Dulbecco’s Modified Eagle Medium (DMEM), dimethyl isosorbide (DMI) (CAS N. 5306-85-4), deuterated dimethylsulfoxide (DMSO-D6), deuterated chloroform (CDCl_3_), acetone (ACS reagent), and thymine were purchased from VWR (Radnor, PA, USA) and used without further purification.

### 3.1. Synthesis and Characterization of Thymine Biomimetic Dimer ***4a-d***

The synthesis of compounds **4a**-**d** was performed by two reaction steps encompassing silylation of thymine **1** followed by alkylation of the corresponding silyl derivative with the appropriate alkyl dibromide. Silylation was performed by a slight modification of previously reported procedures [22,25,26]. Briefly, thymine **1** (4.76 mmol) was dissolved in dry CH_3_CN (5.0 mL) and *N*,*O*-bis-(trimethylsilyl)-acetamide (BSA) (16.32 mmol, 4.0 mL) at 25 °C for 4.0 h. At the end, the mixture was evaporated under reduced pressure to yield *O*,*O*-bis-trimethylsilyl thymine **2** in quantitative yield. Compound **2** (4.76 mmol) was dissolved in the appropriate alkyl-dibromide (2.37 mmol) and dry piperidine (0.05 mmol; 5.0 μL) at 80 °C under argon atmosphere for 4 days. Compounds **4a**-**d** were purified by flash-chromatography, and their purity was analyzed by HPLC as follows: **4a**-**d** (0.05 mg/mL) in CH_3_CN (1.0 mL) were analyzed by an Ultimate 3000 Rapid Resolution UHPLC system (ThermoFisher Scientific) equipped with an Alltima C18 (250 mm × 4.6 mm, 5 mm) column and multi-wavelength detector (254, 271, 280, 333, 335, 600, 720 nm) using 20% CH_3_CN and ultra-pure water 80% as eluent (run 80 min). The following retention times were measured: **4a**, 57.47 min; **4b**, 45.36 min; **4c**, 51.24 min; **4d**, 56.94 min. The NMR spectra were reported in Figure S4.

*N*1,*N*1′-(propane-1,3-diyl)bis-thymine **4a**. Yield: 85% (1.2 g); mp: 330–334 °C, white solid. Elemental analysis for C_13_H_16_N_4_O_4_, expected value: C, 53.42; H, 5.52; N, 19.17; O, 21.89; found C, 53.41; H, 5.50; N, 19.17; O, 21.89. MS (EI, 70 eV) *m*/*z* 292.12. ^1^H NMR (400 MHz, DMSO-d6): δ (ppm) 11.20 (broad s., 2H, NH), 7.52 (s, 2H, H-6), 3.65 (t, 4H, J 7.04 Hz, N-CH_2_), 1.91 (m, 2H, J 7.04 Hz, CH_2_), 1.74 (s, 6H, CH_3_). ^13^C NMR (150 MHz, DMSO-d6): δ (ppm) 162.72 (CO), 149.36 (CO), 137.68 (CH), 105.98 (C), 42.27 (CH_2_), 24.81 (CH_2_), 8.37 (CH_3_).

*N*1,*N*1′-(butane-1,4-diyl)bis-thymine **4b**. Yield: 89.6% (1.3 g), mp: 348–350 °C, brown solid. Elemental analysis for C_14_H_18_N_4_O_4_, expected value: C, 54.89; H, 5.92; N, 18.29; O, 20.89; found C, 54.88; H, 5.87; N, 18.29; O, 20.89. MS (EI, 70 eV) *m*/*z* 306.13. ^1^H NMR (400 MHz, DMSO-d6): δ (ppm) 11.22 (broad s, 2H, NH), 7.53 (s, 2H, H-6), 3.55 (m, 4H, N-CH_2_), 2.32 (s, 6H, CH_3_), 1.75 (m, 4H, CH_2_); ^13^C NMR (150 MHz, DMSO-d6): δ 161.0 (CO), 147.2 (CO), 138.0 (CH), 105.0 (C), 43.0 (CH_2_), 19.84 (CH_2_), 8.37 (CH_3_).

*N*1,*N*1′-(pentane-1,5-diyl)bis-thymine **4c**. Yield: 83.5% (1.3 g), mp: 250–252 °C, white solid. Element analysis for C_15_H_20_N_4_O_4_, expected value: C, 56.24; H, 6.29; N, 17.49; O, 19.98; found C, 56.33; H, 6.30; N, 17.49; O, 19.98. MS (EI, 70 eV) *m*/*z* 320.15. ^1^H NMR (400 MHz, DMSO-d6): δ (ppm) 11.32-10.59 (broad s, 2H, NH), 7.51 (s, 2H, H-6), 3.60 (m, 4H, N-CH_2_), 2.50 (s, 6H, CH_3_), 1.57 (m, 4H, CH_2_), 1.24 (m, 2H, CH_2_); ^13^C NMR (150 MHz, DMSO- d6): δ 160.60 (CO), 147.19 (CO), 137.75 (CH), 104.71 (C), 43.27 (CH_2_), 24.38 (CH_2_), 19.02 (CH_2_), 8.25 (CH_3_).

*N*1,*N*1′-(hexane-1,6-diyl)bis-thymine **4d**. Yield: 68% (1.1 g), mp: 233–235 °C, white solid. Element analysis for C_16_H_22_N_4_O_4_, expected value: C, 57.47; H, 6.63; N, 16.76; O, 19.14; found C, 57.50; H, 6.71; N, 16.76; O, 19.14. MS (EI, 70 eV) *m*/*z* 334.16. ^1^H NMR (400 MHz, DMSO-d6): δ (ppm) 11.18 (broad s, 2H, NH), 7.52 (s, 2H, H-6), 3.59 (m, 4H, N-CH_2_), 2.33 (s, 6H, CH_3_), 1.55 (m, 4H, CH_2_), 0.66 (m, 4H, CH_2_); ^13^C NMR (150 MHz, DMSO-d6): δ 160.62 (CO), 147.25 (CO), 137.78 (CH), 104.79 (C), 43.05 (CH_2_), 25.73 (CH_2_), 21.84 (CH_2_), 8.28 (CH_3_).

*N*1-(3-bromopropyl)-thymine **5a**. Yield: 8.7% (101.9 mg), mp: 240–242 °C, white solid. Elemental analysis for C_8_H_11_BrN_2_O_2_, expected value: C, 38.89; H, 4.49; Br, 32.34; N, 11.34; O, 12.95; found C, 38.78; H, 4.51; Br, 32.34; N, 11.34; O, 12.95. MS (EI, 70 eV) *m*/*z* 246 (100%), 248 (97%). ^1^H NMR (400 MHz, DMSO-d6): δ (ppm) 11.32 (broad s., 1H, NH), 7.19 (s, 1H, H-6), 3.68 (t, 2H, N-CH_2_), 3.51 (t, 2H, CH_2_), 2.34 (s, 3H, CH_3_), 2.06 (m, 2H, CH_2_). ^13^C NMR (150 MHz, DMSO-d6): δ (ppm) 163.7 (CO), 148.8 (CO), 139.2 (CH), 106.9 (C), 48.9 (CH_2_), 37.9 (CH_2_), 30.0 (CH_2_), 9.1 (CH_3_).

*N*1-(4-bromobutyl)-thymine **5b**. MW: 261.12, Yield: 10.4% (128.7 mg), mp: 250–253 °C, brown solid. Element analysis for C_9_H_13_BrN_2_O_2_, expected value: C, 41.40; H, 5.02; N, 10.73; O, 12.25; found C, 41.43; H, 5.01; N, 10.73; O, 12.25. MS (EI, 70 eV) *m*/*z* 260.02 (100.0%), 262.01 (97.3%). ^1^H NMR (400 MHz, DMSO-d6): δ (ppm) 11.32 (broad s., 1H, NH), 7.29 (s, 1H, H-6), 3.68 (m, 2H, N-CH_2_), 3.52 (m, 2H, CH_2_), 2.34 (s, 3H, CH_3_), 1.82 (m, 2H, CH_2_), 1.52 (m, 2H, CH_2_). ^13^C NMR (150 MHz, DMSO-d6): δ (ppm) 169.7 (CO), 150.8 (CO), 139.2 (CH), 110.9 (C), 44.5 (CH_2_), 33.4 (CH_2_), 23.7 (CH_2_), 20.1 (CH_2_), 12.4 (CH_3_).

*N*1-(5-bromopentyl)-thymine **5c**. Yield: 16.5% (215.2 mg), mp: 263–265 °C, white solid. Element analysis for C_10_H_15_BrN_2_O_2_, expected value: C, 43.65; H, 5.50; N, 10.18; O, 11.63; found C, 43.71; H, 5.47; N, 10.18; O, 11.63. MS (EI, 70 eV) *m*/*z* 274.03 (100.0%), 276.03 (97.3%). ^1^H NMR (400 MHz, DMSO-d6): δ (ppm) 11.32 (broad s., 1H, NH), 7.59 (s, 1H, H-6), 3.58 (m, 2H, N-CH_2_), 3.52 (m, 2H, CH_2_), 2.34 (s, 3H, CH_3_), 1.82 (m, 2H, CH_2_), 1.63 (m, 2H, CH_2_) 1.29 (m, 2H, CH_2_). ^13^C NMR (150 MHz, DMSO-d6): δ (ppm) 163.7 (CO), 148.8 (CO), 139.2 (CH), 110.9 (C), 50.1 (CH_2_), 33.7 (CH_2_), 32.2 (CH_2_), 29.3 (CH_2_), 25.1 (CH_2_), 12.4 (CH_3_).

*N*1-(6-bromohexyl)-thymine **5d**. Yield: 32% (438.8 mg), mp: 268–276 °C, white solid. Element analysis for C_11_H_17_BrN_2_O_2_, expected value: C, 45.69; H, 5.93; N, 9.69; O, 11.07; found C, 45.70; H, 5.94; N, 9.69; O, 11.07. MS (EI, 70 eV) *m*/*z* 288.05 (100.0%), 290.05 (97.3%). ^1^H NMR (400 MHz, DMSO-d6): δ (ppm) 11.32 (broad s., 1H, NH), 7.59 (s, 1H, H-6), 3.68 (m, 2H, N-CH_2_), 3.52 (m, 2H, Br-CH_2_), 2.34 (s, 3H, CH_3_), 1.82 (m, 2H, CH_2_), 1.63 (m, 2H, CH_2_) 1.29 (m, 4H, CH_2_). ^13^C NMR (150 MHz, DMSO-d6): δ (ppm) 163.7 (CO), 149.1 (CO), 139.2 (CH), 106.9 (C), 50.2 (CH_2_), 33.7 (CH_2_), 32.6 (CH_2_), 30.3 (CH_2_), 27.7 (CH_2_), 25.7 (CH_2_), 12.4 (CH_3_).

### 3.2. Synthesis of Photo-Adducts CPDs ***6a-d***, (6-4)PPs ***7a-d***, and DVs ***8a-d***

Biomimetic thymine dimers **4a**-**d** (0.20 mmol) were dissolved in MilliQ water–acetone mixtures (from 20% to 50% *v*/*v*, 100 mL) in a Pyrex flask furnished in a refrigerator, deaerated by high-purity Argon (>99.999% purity) for 20 min, and irradiated with a Haerus source (250 Watt, 5 cm distance between flask and lamp) for 2 h under argon atmosphere at the selected temperature (25 °C or, in alternative, 0 °C). The reaction was purified by flash-chromatography (CH_2_Cl_2_/MeOH 9.8:0.2) to yield CPDs **6a**-**d**, (6-4)PPs **7a**-**d**, and DVs **8a**-**d**. The products were characterized by HPLC, UV–visible, and ^1^H-NMR and ^13^C-NMR analyses. For the HPLC analysis, the appropriate compound (0.05 mg/mL) was solubilized in CH_3_CN (1 mL) and separated by an Ultimate 3000 Rapid Resolution UHPLC system (Thermo Fisher Scientific Waltham, MA, USA) equipped with an Altama C18 (250 mm × 4.6 mm, 5 mm) column and multi-wavelength detector (191, 192, 254, 271, 280, 335, 600 nm) using 20% CH_3_CN and ultra-pure water 80% as eluent (run 80 min). The following retention times were measured: **6a**, 57.1; **6b**, 57.3; **6c**, 58.1; **6d**, 58.3; **8a**, 56.8; **8b**, 58.1; **8c**, 58.6; **8d**, 59.1. CPD **6a**. The UV–visible band at 271 nm (C=C) disappeared as a consequence of the cycloaddition process. The NMR spectra were reported in Figure S4.

6a,6b-dimethylhexahydro-1H-3a,5,8,9a-tetraazacyclohepta[*def*]biphenylene 4,6,7,9(5H,8H)-tetraone **6a** was recovered as a mixture of TS and CS isomers. Yield: 31% (18.1 mg); oil. Elemental analysis for C_13_H_16_N_4_O_4_, expected value: C, 53.42; H, 5.52; N, 19.17; O, 21.89; found C, 53.40; H, 5.48; N, 19.17; O, 21.89. MS (EI, 70 eV) *m*/*z* 292.12. ^1^H NMR (400 MHz, CDCl_3_): δ (ppm) 4.40-4.39 (d, 1H, J 8.5 Hz, H-6; TS), 4.36 (s, 1H, H-6, CS) 4.29-4.27 (d, 1H, J 8.5 Hz, H-6; TS), 3.90-3.82 (m, 4H, N-CH_2_), 2.0-1.98 (m, 2H, CH_2_), 1.25 (s, 6H, CH_3_). ^13^C NMR (150 MHz, DMSO-d6): δ (ppm) 157.0 (CO), 152.0 (CO), 63.7 (CH), 51.2 (C), 43.50 (N-CH_2_), 22.60 (CH_2_), 15.8 (CH_3_).

7a,7b-dimethyloctahydro-4a,6,9,10a-tetraazacycloocta[def]biphenylene-5,7,8,10(6H,9H)-tetraone **6b**. Recovered as a mixture of TS and CS isomers. Yield: 33% (20.2 mg); oil. Elemental analysis for C_14_H_18_N_4_O_4_, expected value: C, 54.89; H, 5.92; N, 18.29; O, 20.89; C, 54.91; H, 5.90; N, 18.29; O, 20.89. MS (EI, 70 eV) *m*/*z* 306.13 (100.0%). ^1^H NMR (400 MHz, CDCl_3_): δ (ppm) 4.50 (m, 4H, N-CH_2_), 4.42 (s, 1H, H-6, CS), 4.36 (d, 1H, J 6.8 Hz, H-6; TS), 4.10 (d, 1H, J 6.8 Hz, H-6; TS), 2.41-2.28 (m, 4H, CH_2_), 1.02 (s, 3H, CH_3_), 1.0 (s, 3H, CH_3_). ^13^C NMR (150 MHz, DMSO-d6): δ (ppm) 160.0 (CO), 154.0 (CO), 64.34 (CH), 49.39 (C), 42.36 (N-CH_2_), 22.14 (CH_2_), 19.14 (CH_3_).

8a,8b-dimethyloctahydro-1H-5a,7,10,11a-tetraazacyclonona[*def*]biphenylene-6,8,9,11(7H,10H)-tetraone **6c**. Recovered as a mixture of TS and CS isomers. Yield: 38% (24.3 mg); oil. Elemental analysis for C_15_H_20_N_4_O_4_, expected value: C, 56.24; H, 6.29; N, 17.49; O, 19.98; found C, 56.27; H, 6.32; N, 17.49; O, 19.98. MS (EI, 70 eV) *m*/*z* 320.15. ^1^H NMR (400 MHz, CDCl_3_): δ (ppm) 4.91 (dd, 1H, J 6.9 Hz, H-6; TS), 4.66 (s, 1H, H-6; CS), 4.35 (dd, 1H, J 6.9 Hz, H-6; TS), 3.79-3.58 (m, 4H, N-CH_2_), 1.90-1.78 (m, 4H, CH_2_), 1.58-140 (m, 2H, CH_2_), 1.25 (s, 3H, CH_3_), 1.23 (s, 3H, CH_3_). ^13^C NMR (150 MHz, DMSO-d6): δ (ppm) 163.2 (CO), 149.6 (CO), 59.5 (CH), 51.1 (C), 50.3 (N-CH_2_), 25.0 (CH_2_), 20.3 (CH_2_), 10.0 (CH_3_).

9a,9b-dimethyldecahydro-6a,8,11,12a-tetraazacyclodeca[*def*]biphenylene-7,9,10,12(8H,11H)-tetraone **6d**. Recovered as a mixture of TS and CS isomers. Yield: 39% (26.1 mg); oil. Elemental analysis for C_16_H_22_N_4_O_4_, expected value: C, 57.47; H, 6.63; N, 16.76; O, 19.14; found C, 57.50; H, 6.67; N, 16.76; O, 19.14. MS (EI, 70 eV) *m*/*z* 334.16. ^1^H NMR (400 MHz, CDCl_3_): δ (ppm) 4.41 (d, 1H, J 5.4 Hz, H-6; TS), 4.19 (d, 1H, J 5.4 Hz, H-6; TS), 3.93 (s, 1H, H-6; CS), 3.69- 3.50 (m, 4H, N-CH_2_), 2.10 (m, 4H, CH_2_), 1.71-1.61 (m, 4H, CH_2_), 1.07 (s, 3H, CH_3_), 1.03 (s, 3H, CH_3_). ^13^C NMR (150 MHz, DMSO-d6): δ (ppm) 163.2 (CO), 149.6 (CO), 53.5 (CH), 47.0 (C), 43.5 (CH_2_), 25.3 (CH_2_), 21.3 (CH_2_), 10.0 (CH_3_).

4-hydroxy-4,6-dimethyl-4,4a,10,11-tetrahydro-1H,9H-5,8-(azenomethano)pyrimido[1,6-e][1,5]diazonine-1,3,13(2H)-trione, **7a**. Yield: 17% (9.3 mg); oil. Elemental analysis for C_13_H_14_N_4_O_3_, expected value: C, 56.93; H, 5.15; N, 20.43; O, 17.50; found C, 56.90; H, 5.12; N, 20.43; O, 17.50. MS (EI, 70 eV) *m*/*z* 274.11 (100%). ^1^H NMR (400 MHz, CDCl_3_): δ (ppm) 7,48 (s, 1H, CH), 5.32 (s, 1H, CH), 4.6-4.47 (m, 4H, N-CH_2_), 3.65 (m, 1H, CH), 2.19 (s, 3H, CH_3_), 2.0 (m, 2H, CH_2_), 1.30 (s, 3H, CH_3_). ^13^C NMR (150 MHz, DMSO-d6): δ (ppm) 172,9 (CO), 164.6 (C) 155.7 (CO), 153.1 (CO), 136.7 (CH), 103.8 (C), 83.4 (C), 63.4 (CH), 59.5 (CH_2_), 57.2 (CH_2_), 28.2 (CH_2_), 19.7 (CH_3_), 13.4 (CH_3_).

4-hydroxy-4,6-dimethyl-4,4a,9,10,11,12-hexahydro-1H-5,8-azenomethano)pyrimido[1,6-a][1,6]diazecine-1,3,14(2H)-trione, **7b**. Yield: 26% (15.0 mg); oil. Elemental analysis for C_14_H_16_N_4_O_3_, expected value: C, 58.32; H, 5.59; N, 19.43; O, 16.65; found C, 58.38; H, 5.62; N, 19.43; O, 16.65. MS (EI, 70 eV) *m*/*z* 288.12 (100.0%). ^1^H NMR (400 MHz, CDCl_3_): δ (ppm) 7.80 (s, 1H, CH), 4.25-4.50 (m, 4H, N-CH_2_), 4.42 (s, 1H, CH), 2.48-2.16 (m, 4H, CH_2_), 2.20 (s, 3H, CH_3_), 1.0 (s, 3H, CH_3_). ^13^C NMR (150 MHz, DMSO-d6): δ (ppm)172.9 (CO), 164.6 (C), 155.1 (CO), 150.8 (CO), 136.7 (CH), 103.8 (C), 83.4 (C), 57.4 (CH), 52.1 (CH_2_), 49.8 (CH_2_), 22.6 (CH_2_), 22.6 (CH_2_), 19.7 (CH_3_), 13.9 (CH_3_).

4-hydroxy-4,6-dimethyl-4,4a,10,11,12,13-hexahydro-1H,9H-5,8-(azenomethano)pyrimido[1,6-a][1,6]diazacycloundecine-1,3,15(2H)-trione, **7c**. Yield: 16% (10.2 mg); oil. Elemental analysis for C_15_H_20_N_4_O_4_, expected value: C, 56.24; H, 6.29; N, 17.49; O, 19.98; found C, 56.30; H, 6.32; N, 17.49; O, 19.98. MS (EI, 70 eV) *m*/*z* 320.15 (100.0%). ^1^H NMR (400 MHz, CDCl_3_): δ (ppm) 7.02 (s, 1H, CH), 4.38 (s, 1H, CH), 3.51-3.24 (m, 4H, N-CH_2_), 2.20 (s, 3H, CH_3_), 1.87-1.72 (m, 4H, CH_2_), 1.54-1.37 (m, 2H, CH_2_), 0.90 (s, 3H, CH_3_). ^13^C NMR (150 MHz, DMSO-d6): δ (ppm)172.9 (CO), 164.4 (C), 155.1 (CO), 152.8 (CO), 136.7 (CH), 103.8 (C), 83.4 (C),63.4 (CH), 52.4 (CH_2_), 50.4 (CH_2_), 27.1 (CH_2_), 25.2 (CH_2_), 20.6 (CH_2_), 19.7 (CH_3_), 13.9 (CH_3_).

4-hydroxy-4,6-dimethyl-4a,7,10,11-tetrahydro-9H-5,7,8-(epinitrilomethano)pyrimido[1,6-e][1,5]diazonine-1,3,13(2H,4H)-trione **8a.** Yield: 28% (15.5 mg); oil. Elemental analysis for C_12_H_14_N_4_O_4_, expected value: C, 51.80; H, 5.07; N, 20.13; O, 23.00; found C, 51.67; H, 5.03; N, 20.13; O, 23.00. MS (EI, 70 eV) *m*/*z* 278.10. ^1^H NMR (400 MHz, CDCl_3_): δ (ppm) 6.92 (s, 1H, CH), 5.31 (s, 1H, CH), 4.6-4.0 (m, 4H, N-CH_2_), 1.98 (m, 2H, CH_2_), 1.56 (s, 3H, CH_3_), 1.27 (s, 3H, CH_3_). ^13^C NMR (150 MHz, DMSO-d6): δ (ppm) 172.9 (CO), 158.8 (CO), 152.8 (CO), 130.6 (C), 109.7 (C), 84.0 (C), 78.4 (CH), 71.4 (CH), 49.6 (CH_2_), 47.7 (CH_2_), 27.8 (CH_2_), 19.6 (CH_3_), 14.0 (CH_3_).

4-hydroxy-4,6-dimethyl-4a,7,9,10,11,12-hexahydro-5,7,8-(epinitrilomethano)pyrimido[1,6-a][1,6]diazecine-1,3,14(2H,4H)-trione **8b.** Yield: 21% (12.9 mg); oil. Elemental analysis for C_14_H_18_N_4_O_4_, expected value: C, 54.89; H, 5.92; N, 18.29; O, 20.89; found C, 54.86; H, 5.89; N, 18.29; O, 20.89. MS (EI, 70 eV) *m*/*z* 306.13 (100.0%). ^1^H NMR (400 MHz, CDCl_3_): δ (ppm) 6.92 (s, 1H, CH), 5.97 (s, 1H, CH), 4.60 (s, 1H, CH), 3.78-3.58 (m, 4H, N-CH_2_), 1.98-1.79 (m, 6H, CH_2_), 1.56 (s, 3H, CH_3_), 1.27 (s, 3H, CH_3_). ^13^C NMR (150 MHz, DMSO-d6): δ (ppm) 172.9 (CO), 158.8 (CO), 152.8 (CO), 130.2 (C), 109.1 (C), 84.0 (C), 77.8 (CH), 65.9 (CH), 52.2 (CH_2_), 50.0 (CH_2_), 22.4 (CH_2_), 22.1 (CH_2_), 19.6 (CH_3_), 14.0 (CH_3_).

4-hydroxy-4,6-dimethyl-4a,7,10,11,12,13-hexahydro-9H-5,7,8-(epinitrilomethano)pyrimido[1,6-a][1,6]diazacycloundecine-1,3,15(2H,4H)-trione **8c**. Yield: 14% (9.0 mg); oil. Elemental analysis for C_15_H_20_N_4_O_4_, expected value: C, 56.24; H, 6.29; N, 17.49; O, 19.98; found C, 56.20; H, 6.32; N, 17.49; O, 19.98. MS (EI, 70 eV) *m*/*z* 320.15 (100.0%). ^1^H NMR (400 MHz, CDCl_3_): δ (ppm) 5.64 (s, 1H, CH), 4.59 (s, 1H, CH), 3.58-3.47 (m, 4H, N-CH_2_), 1.90-1.78 (m, 4H, CH_2_), 1.54-1.37 (m, 2H, CH_2_), 1.21 (s, 3H, CH_3_), 0.90 (s, 3H, CH_3_). ^13^C NMR (150 MHz, DMSO-d6): δ (ppm) 172.9 (CO), 158.8 (CO), 152.8 (CO), 130.2 (C), 109.1 (C), 84.3 (C), 77.8 (CH), 71.9 (CH), 52.5 (CH_2_), 50.3 (CH_2_), 20.3 (CH_2_), 20.1 (CH_2_), 19.6 (CH_3_), 14.0 (CH_3_).

**8d**. Yield: 23% (15.4 mg); oil. Elemental analysis for C_16_H_22_N_4_O_4_, expected value: C, 57.47; H, 6.63; N, 16.76; O, 19.14; found C, 57.50; H, 6.60; N, 16.76; O, 19.14. MS (EI, 70 eV) *m*/*z* 334.16 (100.0%). ^1^H NMR (400 MHz, CDCl_3_): δ (ppm) 5.55 (s, 1H, CH), 4.28 (s, 1H, CH), 3.89-3.60 (m, 4H, N-CH_2_), 1.53-1.45 (m, 4H, CH_2_), 1.26-1.11 (m, 4H, CH_2_), 1.19 (s, 3H, CH_3_), 0.98 (s, 3H, CH_3_). ^13^C NMR (150 MHz, DMSO-d6): δ (ppm) 172.9 (CO), 158.8 (CO), 152.8 (CO), 130.2 (C), 109.1 (C), 84.3 (C), 77.8 (CH), 65.9 (CH), 52.5 (CH_2_), 50.3 (CH_2_), 29.1 (CH_2_), 29.1 (CH_2_), 21.1 (CH_2_), 21.1 (CH_2_), 19.6 (CH_3_), 14.0 (CH_3_).

### 3.3. Preparation and Loading of Lignin Nanoparticles

LNPs were produced and loaded by the nanoprecipitation technique. The appropriate compound (3 mg) was dissolved in DMI/water (3.0 mL) at 50 °C under gentle stirring conditions followed by the addition of KL (15 mg). The fast addition of deionized water (6.0 mL) instantaneously produced LNPs. LNPs were then recovered by centrifugation (7500 rpm), washed with deionized water (10 mL) for 3 times, and lyophilized. The loading capacity (LC) and loading efficiency (LE) were evaluated by UHPLC analysis.

### 3.4. UV Photo-Protective Effect of Loaded LNPs

LNPs/**4a** were prepared following the standard procedure previously described. Typically, LNPs/**4a** (1.0 mg) suspended in DMI/water mixture (2:1 ratio, 1.0 mL) were deposited on a glass lens and irradiated with Haerus source (250 Watt, the lamp was at 20.0 cm distance). At scheduled time intervals (5, 10, and 15 min), 0.2 mL of irradiated colloidal suspension were withdrawn and treated with DMI (0.2 mL) to disaggregate LNP structures, allowing the recovery of residual **4a**. The photo-protection capacity of LNPs was determined by measuring the decrease in absorbance (λ_max_ 271 nm) of recovered **4a** compared with free reference (Varian UVCary-50). The irradiation of **4a** (1.0 mg) alone, under similar experimental conditions, was performed as a reference.

### 3.5. Kinetic Release of Lignin Nanoparticles

LNPs (7.5 mg, compound/KL 1:5 ratio) were suspended in water (3.0 mL), placed in a dialysis membrane, and immersed in PBS buffer (60 mL) at two selected pH values (7.4 and 5.5, respectively) at 37.5 °C for 24 h. A similar procedure was applied solubilizing LNPs/**8b** (7.5 mg) water (3.0 mL), followed by dialysis against the culture medium DMEM (60 mL). At scheduled time intervals (2, 5, 10, 15, 20, 30, 45, 60, 120, 180 min and 24 h) aliquots of the solution (0.2 mL) were withdrawn for UV–visible analysis, the same volume of fresh medium being continuously replaced to maintain constant the total volume of the system. The quantification of released compound was conducted by UV–Vis spectrophotometry at the λ_max_ characteristic for each type of compound (271 nm for **4a**-**d**, and 191 and 192 nm for **6a**-**d** and **8a**-**d**, respectively) using semi-micro cuvettes. Water (1.5 mL) was used as a baseline reference.

### 3.6. Biological Assay

FB789 was grown in DMEM/F10, RPMI7951 melanoma cancer cells were grown in Minimal Essential Medium (MEM), while SK-Mel 28 melanoma cancer cells were grown in Dulbecco’s Modified Eagle Medium (DMEM). All culture media were supplemented with 10% Fetal Bovine Serum (FBS) plus 1.0 mM glutamine (40 µg/mL). The antimelanoma activity of **4a**-**d**, **6a**-**d**, and **8a**-**d** was evaluated by MTT viability assay according to reference [87]. Briefly, aliquots of the appropriate compound (1.0 mmol) were solubilized in dimethyl sulfoxide (one drops, DMSO) and administered to the selected cell line. After incubation for 3 h at 37 °C with MTT (0.5 mg/mL) the supernatant was removed, and 100 mL of lysis solution (10% SDS and 0.6% acetic acid) was added to dissolve the formazan crystals. Optical density detection was performed by DTX880 Multimode Detector (Beckman Coulter) with 630 nm (background) and 570 nm filters. The percentage of cell viability was calculated as follows: % cell viability = 100–% cell cytotoxicity. The experiments for the evaluation of the antimelanoma activity of LNPs/**8a** and LNPs/**8b** were performed in a similar way using the appropriate amount of nanoparticles containing 1.0 mmol of the active compound and analyzing the antimelanoma activity at three different release times (2, 4, and 24 h).

### 3.7. In Silico Molecular Docking Analysis

The crystal structure of TOP1 and 22 base pair DNA duplex (PDB ID: 1T8I) was used in order to perform molecular docking analysis. The energy minimization run of TOP1/DNA duplex was performed by the steepest descent algorithm with a maximum number of minimization steps of 50,000. The complex was centered in a dodecahedron box with a minimal distance of 1.0 nm to the edge of the box, and the TIP3P water model was used to solvate the system by adding 18 sodium ions [88]. Both the receptor and the ligands were prepared for the docking analysis using Autodocktools v. 1.5.6 [89]. The receptor was prepared by removing crystal ligands, adding polar hydrogens and Kollman charges as partial charges. 3D coordinates of **8a**, **8b**, **4a**, and **4b**, provided in smile format, were generated by using Open Babel software v. 2.3.2 [90]. Polar hydrogens were added to each ligand, and Gasteiger charges were added as partial charges.

AutoDock Vina software [91] was used to perform docking experiments between the selected compound and TOP1/DNA duplex. A grid box with size 10 × 10 × 10 and centered at X = 94.906 Y = 95.914 Z = 32.500 was used as search space. AutoDock Vina provided the best binding affinity rank for each ligand.

## 4. Conclusions

A panel of biomimetic thymine dimers and photo-adducts, differing in the number of carbon atoms in the spacer between nucleobases, was synthesized under photochemical conditions. The highest yield of photo-adducts was obtained in the presence of acetone as photosensitizer at 0 °C. The complete panel of expected products was isolated, including CPD, (6-4)PP and DV photo-adducts. In the case of CPD, the steric constrains of the linker favored the formation of the trans-syn stereoisomer with respect to the cis-syn counterpart. CPD were synthesized in a yield higher than the other products. Due to the reported affinity between pyrimidine photo-adducts and TOP1, the antimelanoma activity of novel derivatives was tested against two mutated cancer cell lines overexpressing TOP1, namely SKMEL-28 and RPMI7951. Among photo-adducts, DV showed higher antimelanoma activity than corresponding (6-4)PP and CPD counterparts, the highest value of SI being associated with compounds **8a** and **8b**. The SI value of these compounds was higher, or of the same order of magnitude, than commercial antitumor agents, such as doxorubicin (SI 26.0), 5-fluro uracil 5-FU (SI 55.6), and camptothecin (SI 88.3) [92]. The antimelanoma activity of **8a** and **8b** was retained after loading inside LNPs, which were able to release gradually the bioactive compound in the culture medium, preserving it from photo-degradation. The encapsulation of compounds **8a** and **8b** was obtained by a sustainable nanoprecipitation procedure affording high loading capacity and efficiency values, as well as optimal kinetic release. Molecular docking analysis suggested the antimelanoma activity of **8a** and **8b** is correlated to the formation of a stable interaction with TOP1/DNA complex at the cleavage site of the system. Overall, these results open a new entry for the design of a sustainable drug-delivery system combining the photo-protective effect of LNP to the antimelanoma activity of thymine photo-adducts produced after the release of thymine dimers on the skin. The possibility that thymine photo-adducts may also activate specific alert pathways for the cell against UV damage represent a further frontier to be investigated.

## Figures and Tables

**Table 1:** Biomimetic thymine dimers produced by alkylation of *O,O*-bis-trimethylsilyl thymine **2** with di-alkyl bromides.

Entry	Conditions ^a^	Product(s)	Spacer ^b^	Conversion (%)	Yield (%)
1	A	**4a** (**5a**)	3	14	22 (78)
2	B	**4a** (**5a**)	3	>99	85 (8.7)
3	C	**4b** (**5b**)	4	>99	89.6 (10.4)
4	D	**4c** (**5c**)	5	>99	83.5 (16.5)
5	E	**4d** (**5d**)	6	>99	68 (32)

^a^ General reaction conditions: compound **2** and di-alkyl bromide (2.37 mmol). A: 1,3-dibromopropane, DMF (5.0 mL), 170 °C. B: 1,3-dibromopropane, piperidine (5.0 μL, 0.05 mmol), 110 °C. C: 1,4-dibromobutane, piperidine (5.0 μL, 0.05 mmol), 110 °C. D: 1,5-dibromopentane, piperidine (5.0 μL, 0.05 mmol), 110 °C. E: 1,6-dibromohexane, piperidine (5.0 μL, 0.05 mmol), 110 °C. ^b^ Number of carbon atoms contained in the spacer connecting thymine nucleobases.

**Table 2:** CPD, (6-4)PP, and DV photo-adducts produced by UV-irradiation of biomimetic thymine dimers **4a**-**d**.

Entry	Substrate	Condition	Conversion (%)	TS:CS ratio ^a^	Product(s) (%)	Yield (%)
1	**4a**	A	>99%	4.0:1.0	**6a** (**7a**) [**8a**]	20 (12) [15]
2	**4a**	B	>99%	4.3:0.9	**6a** (**7a**) [**8a**]	26 (16) [19]
3	**4a**	C	>99%	4.2:0.9	**6a** (**7a**) [**8a**]	31 (17) [28]
4	**4b**	C	>99%	3.1:1.0	**6b** (**7a**) [**8a**]	33 (26) [21]
5	**4c**	C	>99%	4.0:1.0	**6c** (**7a**) [**8a**]	38 (16) [14]
6	**4d**	C	>99%	3.5:1.0	**6d** [**8a**]	39 [23]

Irradiation was performed by using Haerus source 250 Watt in a Pyrex flask under argon atmosphere for 2.0 h. A: water:acetone 8:2 *v*/*v* ratio (110 mL), 25 °C. B: water:acetone 8:2 *v*/*v* ratio (110 mL), 0 °C. C: water:acetone 6:4 *v*/*v* ratio (110 mL), 0 °C. ^a^ Ratio between the trans-syn (TS) and cis-syn (CS) isomers in the case of CPD dimers.

**Table 3:** Preparation and loading of biomimetic thymine dimer **4a** into LNPs by nanoprecipitation technology ^a^.

Entry	II Solvent (mL) ^b^	1:5 ^c^	1:10	1:20
1	×1	Precipitate	Precipitate	Precipitate
2	×2	Stable colloid	Stable colloid	Low stable colloid
3	×4	Stable colloid	Stable colloid	Low stable colloid
4	×6	Low stable colloid	Low stable colloid	Low stable colloid
5	×8	Absence of LNPs	Aggregated LNPs	Aggregated LNPs
6	×10	Absence of LNPs	Absence of LNPs	Absence of LNPs
7	×12	Absence of LNPs	Absence of LNPs	Absence of LNPs
8	×14	Absence of LNPs	Absence of LNPs	Absence of LNPs

^a^ General condition for the preparation of LNPs loaded with compound **4a** include 3.0 mL of DMI/water 2:1 *v*/*v* as primary solvent containing **4a** and KL at 25 °C. ^b^ The volume of secondary solvent (deionized water) expressed as multiple of the volume of the primary solvent. ^c^ **4a**/KL *w*/*w* ratio (1:5 *w/w*, 1:10 *w/w*, and 1:20 *w/w* values, respectively).

**Table 4:** Loading capacity and loading efficiency of LNPs in the encapsulation of biomimetic thymine dimers **4a**-**d** and photo-adducts **6a**-**d** and **8a**-**d** ^a^.

Entry	Compound(s)	Compd/KL Ratio	LE%	LC%	Compd/KL Ratio	LE%	LC%
1	**4a**	1:5	49.94	5.59	1:10	49.26	4.93
2	**4b**	1:5	51.91	10.38	1:10	52.40	4.24
3	**4c**	1:5	69.70	12.52	1:10	62.59	6.97
4	**4d**	1:5	71.85	14.37	1:10	71.41	7.14
5	**6a**	1:5	37.40	6.93	1:10	41.12	6.81
6	**6b**	1:5	50.94	9.59	1:10	50.74	9.33
7	**6c**	1:5	51.12	11.46	1:10	47.86	10.48
8	**6d**	1:5	72.61	13.22	1:10	71.98	11.54
9	**8a**	1:5	35.34	6.80	1:10	33.21	6.89
10	**8b**	1:5	52.12	9.65	1:10	50.74	9.45
11	**8c**	1:5	53.23	11.58	1:10	52.86	11.67
12	**8d**	1:5	75.34	13.55	1:10	74.98	74.37

^a^ LC and LE have been determined at two values of compound/KL ratio, 1:5 and 1:10, respectively. Measurements were repeated in triplicate.

**Table 5:** Antimelanoma activity of biomimetic thymine dimers **4a**-**d** and photo-adducts **6a**-**d** and **8a**-**d**.

Type	Entry	Compd(s)	Time (h)	FB789 (CC_50_ µM)	SK-Mel28 (IC_50_ µM)	SI (SKMel28)	RPMI7951 (IC_50_ µM)	SI (RPMI7951)
**Biomimetic** **Dimers**	1	**4a**	24	1450 ± 56.18	304.3 ± 2.4	4.7	543.1 ± 2.7	2.7
	2	**4b**	24	839.2 ± 10.9	169.4 ± 4.6	4.9	12,446 ± 16	0.07
	3	**4c**	24	1662 ± 36.3	292.9 ± 2.5	5.7	356.7 ± 5.1	4.6
	4	**4d**	24	392.5 ± 5.2	169.4 ± 4.6	2.3	342.8 ± 7.6	1.14
**CPDs**	5	**6a**	24	374.9 ± 28.15	85.23 ± 0.9	4.39	138.1 ± 2.1	2.7
	6	**6b**	24	109.1 ± 5.4	17.89 ± 1.9	6.09	77.76 ± 1.8	1.4
	7	**6c**	24	234.3 ± 7.3	282.4 ± 4.4	0.83	195.1 ± 3.8	1.2
	8	**6d**	24	376.8 ± 14.8	87.91 ± 1.9	4.3	234.9 ± 1.6	1.6
**Dewar Valence**	9	**8a**	24	4935 ± 58.7	146.9 ± 2.2	33.6	94.27 ± 1.9	52.3
	10	**8b**	24	12,442 ± 16.05	53.07 ± 1.7	234.4	120.6 ± 2.9	103.2
	11	**8c**	24	250.4 ± 3.4	138.8 ± 2.1	1.8	77.89 ± 1.05	3.2
	12	**8d**	24	33.47 ± 1.4	93.08 ± 1.9	0.36	114.9 ± 6.8	0.29
	13	LNPs/**8a**	2	3454 ± 1.9	129.1 ± 1.9	26.8	77.4 ± 1.8	44.6
			4	4112 ± 1.7	145.4 ± 1.1	28.3	88.2 ± 0.9	46.6
			24	5012.1 ± 2.1	160.8 ± 2.4	31.2	41.1 ± 2.1	47.92
	14	LNPs/**8b**	2	11,783 ± 1.1	59.8 ± 2.1	197.0	129.5 ± 1.7	91.0
			4	13,601 ± 3.1	68.7 ± 0.8	198.0	139.3 ± 1.1	97.6
			24	14,888.1 ± 1.4	69.8 ± 0.9	213.3	148.8 ± 1.4	100.1

Results in terms of IC_50_ and SI after 24 h of treatment with compounds **4a**-**d**, **6a**-**d**, and **8a**-**d** in a normal human fibroblast cell line (FB789) and two melanoma cell lines (SK-Mel28, RMPI7951).

**Table 6:** Docking analysis for the compounds **8a**-**b** and **4a**-**b**.

Entries	Compd(s)	Binding Affinity (Kcal/mol) ^a^	Hydrogen Bonds ^b^	Salt Bridges ^b^	Hydrophobic Interactions ^b^	Π-Cation Interactions ^b^	Π-Stacking Interactions ^b^
1	**8a**	−7.9	DC112, DA113, ARG364, ASP533, THR718	-	-	DA113	-
2	**8b**	−8.8	DC111, DC112, ARG364, ASP533, THR718	ASP533	-	-	-
3	**4a**	−7.1	DG12, DC112, ARG364, THR718	-	DT110, LYS532, THR718	-	DG12
4	**4b**	−7.3	DC112, DA113, ARG364	-	-	-	-

^a^ Docking analysis (AutoDock Vina software); ^b^ Analysis of non-covalent interactions (PLIP software).

## Data Availability

Not applicable.

## Supplementary Materials

The following material is available online at www.mdpi.com/article/10.3390/ijms23020915/s1.

## References

[1] Shen Y., Stanislauskas M., Li G., Zheng D., Liu L. (2017). Epigenetic and genetic dissections of UV-induced global gene dysregulation in skin cells through multi-omics analyses. Sci. Rep..

[2] Ravanat J.L., Douki T., Cadet J. (2001). Direct and indirect effects of UV radiation on DNA and its components. J. Photochem. Photobiol. B.

[3] Cadet J., Mouret S., Ravanat J.L., Douki T. (2012). Photoinduced damage to cellular DNA: Direct and photosensitized reactions. Photochem. Photobiol..

[4] Saha L.K., Wakasugi M., Akter S., Akter S., Prasad R., Wilson S.H., Shimizu N., Sasanuma H., Huang S.N., Agama K. (2020). Topoisomerase I-driven repair of UV-induced damage in NER-deficient cells. Proc. Natl. Acad. Sci. USA.

[5] Cadet J., Wagner J.R. (2013). DNA base damage by reactive oxygen species, oxidizing agents, and UV radiation. Cold Spring Harb. Perspect. Biol..

[6] Napolitano A., Panzella L., Monfrecola G., d’Ischia M. (2014). Pheomelanin-induced oxidative stress: Bright and dark chemistry bridging red hair phenotype and melanoma. Pigment Cell Melanoma Res..

[7] Giglia-Mari G., Zotter A., Vermeulen W. (2011). DNA damage response. Cold Spring Harb. Perspect. Biol..

[8] Athar M., Kim A.L., Ahmad N., Mukhtar H., Gautier J., Bickers D.R. (2000). Mechanism of ultraviolet B-induced cell cycle arrest in G2/M phase in immortalized skin keratinocytes with defective p53. Biochem. Biophys. Res. Commun..

[9] Cooke M.S., Harry E.L., Liljendahl T.S., Segerbäck D. (2013). DNA nucleotide excision repair, where do all the cyclobutane pyrimidine dimers go?. Cell Cycle.

[10] Kemp M.G., Hu J. (2016). PostExcision Events in Human Nucleotide Excision Repair. Photochem. Photobiol..

[11] Cadet J., Douki T. (2018). Formation of UV-induced DNA damage contributing to skin cancer development. Photochem. Photobiol. Sci..

[12] Schuch A.P., Moreno N.C., Schuch N.J., Menck C., Garcia C. (2017). Sunlight damage to cellular DNA: Focus on oxidatively generated lesions. Free Radic. Biol. Med..

[13] Hu J., Choi J.H., Gaddameedhi S., Kemp M.G., Reardon J.T., Sancar A. (2013). Nucleotide excision repair in human cells: Fate of the excised oligonucleotide carrying DNA damage in vivo. J. Biol. Chem..

[14] Kciuk M., Marciniak B., Mojzych M., Kontek R. (2020). Focus on UV-Induced DNA Damage and Repair-Disease Relevance and Protective Strategies. Int. J. Mol. Sci..

[15] Lanza A., Tornaletti S., Rodolfo C., Scanavini M.C., Pedrini A.M. (1996). Human DNA topoisomerase I-mediated cleavages stimulated by ultraviolet light-induced DNA damage. J. Biol. Chem..

[16] Subramanian D., Rosenstein B.S., Muller M.T. (1998). Ultraviolet-induced DNA damage stimulates topoisomerase I-DNA complex formation in vivo: Possible relationship with DNA repair. Cancer Res..

[17] Pommier Y. (2006). Topoisomerase I inhibitors: Camptothecins and beyond. Nat. Rev. Cancer..

[18] Pommier Y., Barcelo J.M., Rao V.A., Sordet O., Jobson A.G., Thibaut L., Miao Z.H., Seiler J.A., Zhang H., Marchand C. (2006). Repair of topoisomerase I-mediated DNA damage. Prog. Nucleic Acid Res. Mol. Biol..

[19] Beiu C., Giurcaneanu C., Grumezescu A.M. (2020). Nanosystems for Improved Targeted Therapies in Melanoma. J. Clin. Med..

[20] Vlasceanu G.M., Victor L., Maricica H., Raluca T., Vlad O., Gheorghe I., Bolocan A., Grumezescu A.M., Holban A.M., Ficai A., Grumezescu A.M. (2017). Nanostructures for cancer therapy: From targeting to selective toxicology. Nanostructures for Cancer Therapy.

[21] Piccinino D., Capecchi E., Tomaino E., Gabellone S., Gigli V., Avitabile D., Saladino R. (2021). Lignin as Green Antioxidant and UV Shielding Ingredient for Sunscreen Applications. Antioxidants.

[22] Raza A., Dreis C.D., Vince R. (2013). Photoprotection of DNA (in vitro) by acyclothymidine dinucleosides. Bioorg. Med. Chem. Lett..

[23] Raza A., Ericson M.E., Nugent J.S., Dreis C.D., Vince R. (2014). A bio-mimetic approach to DNA photoprotection. J. Investig. Dermatol..

[24] Nugent J.S., Vince R., Raza A. (2015). Topical Acyclothymidine Dinucleosides (aTds) Promote Non-UV-Mediated Endogenous Defense Mechanisms in Guinea Pig Skin. J. Investig. Dermatol..

[25] Moidoveanu S.C., David V. (2002). Derivatization Reactions for Analytes with Various Functional Groups. Sample Preparation in Chromatography.

[26] Hanessian S., Saladino R., Nunez J.C. (1996). On the binding site of quinolone antibacterials. An attempt to probe the shen model. Bioorg. Med. Chem. Lett..

[27] Varghese A.J., Wang S.Y. (1968). Thymine-thymine adduct as a photoproduct of thymine. Science.

[28] Varghese A.J. (1970). Photochemistry of thymidine in ice. Biochemistry.

[29] Wagner P.J., Bucheck D.J. (1970). Photodimerization of Thymine and Uracil in Acetonitrile. J. Am. Chem. Soc..

[30] Taylor J.S., Lu H.F., Kotyk J.J. (1990). Quantitative conversion of the (6-4) photoproduct of TpdC to its Dewar valence isomer upon exposure to simulated sunlight. Photochem. Photobiol..

[31] Kohei T., Chikahide M., Fumio H., Shigenori I. (2004). Chemical synthesis and translesion replication of a cis-syn cyclobutane thymine-uracil dimer. Nucleic Acids Res..

[32] Tohnai N., Miyata M., Yasui N., Mochizuki E., Kai K., Inaki Y. (1998). Photodimerization of Thymine Derivatives in Single Crystal. J. Photopolym. Sci. Technol..

[33] Cadet J., Voituriez L., Frank E., Hruska L.S.K., de Leeuw F.A.A.M., Altona A. (1985). Characterization of thymidine ultraviolet photoproducts. Cyclobutane dimers and 5,6-dihydrothymidines. Can. J. Chem..

[34] Gut I.G., Wood P.D., Redmond R.W. (1996). Interaction of triplet photosensitizers with nucleotides and DNA in aqueous solution at room temperature. J. Am. Chem. Soc..

[35] Ramamurthy V., Sivaguru J. (2016). Supramolecular Photochemistry as a Potential Synthetic Tool: Photocycloaddition. Chem. Rev..

[36] Epe B., Henzl H., Adam W., Saha-Moller C.R. (1993). Endonuclease-sensitive DNA modifications induced by acetone and acetophenone as photosensitizers. Nucleic Acids Res..

[37] Kan L.S., Voituriez L., Cadet J. (1992). The Dewar valence isomer of the (6-4) photoadduct of thymidylyl-(3’-5’)-thymidine monophosphate: Formation, alkaline lability and conformational properties. J. Photochem. Photobiol. B.

[38] Nagpal A., Dhankhar D., Cesario T.C., Li R., Chen J., Rentzepis P.M. (2021). Thymine dissociation and dimer formation: A Raman and synchronous fluorescence spectroscopic study. Proc. Natl. Acad. Sci. USA.

[39] Shigenori I., Masato S., Hiroyuki K., Eiko O. (1996). Synthesis of a Phosphoramidite Coupling Unit of the Pyrimidine (6-4) Pyrimidone Photoproduct and Its Incorporation into Oligodeoxynucleotides. J. Am. Chem. Soc..

[40] Yokoyama H., Mizutani R. (2014). Structural biology of DNA (6-4) photoproducts formed by ultraviolet radiation and interactions with their binding proteins. Int. J. Mol. Sci..

[41] Mu W., Han Q., Luo Z., Wang Y. (2006). Production of cis-syn thymine-thymine cyclobutane dimer oligonucleotide in the presence of acetone photosensitizer. Anal. Biochem..

[42] Lu C., Gutierrez-Bayona N.E., Taylor J.S. (2021). The effect of flanking bases on direct and triplet sensitized cyclobutane pyrimidine dimer formation in DNA depends on the dipyrimidine, wavelength and the photosensitizer. Nucleic Acids Res..

[43] Goto N., Bazar G., Kovacs Z., Kunisada M., Morita H., Kizaki S., Sugiyama H., Tsenkova R., Nishigori C. (2015). Detection of UV-induced cyclobutane pyrimidine dimers by near-infrared spectroscopy and aquaphotomics. Sci. Rep..

[44] Jiang Y., Rabbi M., Kim M., Ke C., Lee W., Clark R.L., Mieczkowski P.A., Marszalek P.E. (2009). UVA generates pyrimidine dimers in DNA directly. Biophys. J..

[45] Mouret S., Baudouin C., Charveron M., Favier A., Cadet J., Douki T. (2006). Cyclobutane pyrimidine dimers are predominant DNA lesions in whole human skin exposed to UVA radiation. Proc. Natl. Acad. Sci. USA.

[46] Piccinino D., Capecchi E., Botta L., Bizzarri B.M., Bollella P., Antiochia R., Saladino R. (2018). Layer-by-Layer Preparation of Microcapsules and Nanocapsules of Mixed Polyphenols with High Antioxidant and UV-Shielding Properties. Biomacromolecules.

[47] Alqahtani M.S., Alqahtani A., Kazi M., Ahmad M.Z., Alahmari A., Alsenaidy M.A., Syed S. (2020). Wound-healing potential of curcumin loaded lignin nanoparticles. J. Drug. Deliv. Sci. Technol..

[48] Siddiqui L., Bag J., Seetha, Mittal D., Leekha A., Mishra H., Mishra M., Verma A.K., Mishra P.K., Ekielski A. (2020). Assessing the potential of lignin nanoparticles as drug carrier: Synthesis, cytotoxicity and genotoxicity studies. Int. J. Biol. Macromol..

[49] Li H., Deng Y., Wu H., Ren Y., Qiu X., Zheng D., Li C. (2016). Self-assembly of kraft lignin into nanospheres in dioxane-water mixtures. Holzforschung.

[50] Lee J.H., Park D.Y., Choi I.G., Choi J.W. (2020). Investigation of Molecular Size Effect on the Formation of Lignin Nanoparticles by Nanoprecipitation. Appl. Sci..

[51] Sipponen M.H., Lange H., Crestini C., Henn A., Österberg M. (2019). Lignin for Nano- and Microscaled Carrier Systems: Applications, Trends, and Challenges. ChemSusChem.

[52] Zhang Z., Terrasson V., Guénin E. (2021). Lignin Nanoparticles and Their Nanocomposites. Nanomaterials.

[53] Piccinino D., Capecchi E., Delfino I., Crucianelli M., Conte N., Avitabile D., Saladino R. (2021). Green and Scalable Preparation of Colloidal Suspension of Lignin Nanoparticles and Its Application in Eco-friendly Sunscreen Formulations. ACS Omega.

[54] Parisi N., Paz-Alvarez M., Matts P.J., Lever R., Hadgraft J., Lane M.E. (2016). Topical delivery of hexamidine. Int. J. Pharm..

[55] Dai L., Liu R., Hu L.Q., Zou Z.F., Si C.L. (2017). Lignin Nanoparticle as a Novel Green Carrier for the Efficient Delivery of Resveratrol. ACS Sustain. Chem. Eng..

[56] Alqahtani M.S., Alqahtani A.H., Al-Thabit A., Roni M.A., Syed R. (2019). Novel lignin nanoparticles for oral drug delivery. J. Mater. Chem. B..

[57] Sipponen M.H., Lange H., Ago M., Crestini C. (2018). Understanding Lignin Aggregation Processes. A Case Study: Budesonide Entrapment and Stimuli Controlled Release from Lignin Nanoparticles. ACS Sustain. Chem. Eng..

[58] Wang M., Yang D., Xu Q., Li P., Yi C., Qian Y., Qiu X. (2021). Highly efficient evaporation method to prepare pH-responsive lignin-hollow-nanosphere with controllable size and its application in oral drug delivery. Ind. Crops Prod..

[59] Chen L., Zhou X., Shi Y., Gao B., Wu J., Kirk T.B., Xu J., Xue W. (2018). Green synthesis of lignin nanoparticle in aqueous hydrotropic solution toward broadening the window for its processing and application. Chem. Eng. J..

[60] Andeme Ela R.C., Tajiri M., Newberry N.K., Heiden P.A., Ong R.G. (2020). Double-Shell Lignin Nanocapsules Are a Stable Vehicle for Fungicide Encapsulation and Release. ACS Sustain. Chem. Eng..

[61] Tao J., Chow S.F., Zheng Y. (2019). Application of flash nanoprecipitation to fabricate poorly water-soluble drug nanoparticles. Acta Pharm. Sin. B..

[62] D’Addio S.M., Prud’homme R.K. (2011). Controlling drug nanoparticle formation by rapid precipitation. Adv. Drug Deliv. Rev..

[63] Mishra P.K., Ekielski A. (2019). The Self-Assembly of Lignin and Its Application in Nanoparticle Synthesis: A Short Review. Nanomaterials.

[64] Deng Y., Feng X., Yang D., Yi C., Qiu X. (2012). Pi-Pi stacking of the aromatic groups in lignosulfonates. BioResources.

[65] Davies R.J., Malone J.F., Gan Y., Cardin C.J., Lee M.P., Neidle S. (2007). High-resolution crystal structure of the intramolecular d(TpA) thymine-adenine photoadduct and its mechanistic implications. Nucleic Acids Res..

[66] Di Pasquale N., Marchisio D.L., Barresi A.A., Carbone P. (2014). Solvent Structuring and Its Effect on the Polymer Structure and Processability: The Case of Water-Acetone Poly-ε-caprolactone Mixtures. J. Phys. Chem. B.

[67] Li H., Deng Y., Liu B., Ren Y., Liang J., Qian Y., Qiu X., Li C., Zheng D. (2016). Preparation of Nanocapsules via the Self-Assembly of Kraft Lignin: A Totally Green Process with Renewable Resources. ACS Sustain. Chem. Eng..

[68] Li Y., Qiu X., Qian Y., Xiong W., Yang D. (2017). pH-responsive lignin-based complex micelles: Preparation, characterization and application in oral drug delivery. Chem. Eng. J..

[69] Zhou Y., Han Y., Li G., Yang S., Xiong F., Chu F. (2019). Preparation of targeted lignin-based hollow nanoparticles for the delivery of doxorubicin. Nanomaterials.

[70] Yiamsawas D., Kangwansupamonkon W., Kiatkamjornwong S. (2021). Lignin-based nanogels for the release of payloads in alkaline conditions. Eur. Polym. J..

[71] Patrick M.H. (1977). Studies on thymine-derived UV photoproducts in DNA-I. Formation and biological role of pyrimidine adducts in DNA. Photochem. Photobiol..

[72] Strozyk E., Kulms D. (2013). The role of AKT/mTOR pathway in stress response to UV-irradiation: Implication in skin carcinogenesis by regulation of apoptosis, autophagy and senescence. Int. J. Mol. Sci..

[73] Hegedűs C., Boros G., Fidrus E., Kis G.N., Antal M., Juhász T., Janka E.A., Jankó L., Paragh G., Emri G. (2020). PARP1 Inhibition Augments UVB-Mediated Mitochondrial Changes—Implications for UV-Induced DNA Repair and Photocarcinogenesis. Cancers.

[74] Emmert S., Kobayashi N., Khan S.G., Kraemer K.H. (2000). The xeroderma pigmentosum group C gene leads to selective repair of cyclobutane pyrimidine dimers rather than 6-4 photoproducts. Proc. Natl. Acad. Sci. USA.

[75] Harada Y.N., Shiomi N., Koike M., Ikawa M., Okabe M., Hirota S., Kitamura Y., Kitagawa M., Matsunaga T., Nikaido O. (2020). Postnatal growth failure, short life span, and early onset of cellular senescence and subsequent immortalization in mice lacking the xeroderma pigmentosum group G gene. Mol. Cell. Biol..

[76] Hiramoto T., Matsunaga T., Ichihashi M., Nikaido O., Fujiwara Y., Mishima Y. (1989). Repair of 254 nm ultraviolet-induced (6-4) photoproducts: Monoclonal antibody recognition and differential defects in xeroderma pigmentosum complementation groups A, D, and variant. J. Investig. Dermatol..

[77] Köberle B., Roginskaya V., Wood R.D. (2006). XPA protein as a limiting factor for nucleotide excision repair and UV sensitivity in human cells. DNA Repair.

[78] Nakane H., Takeuchi S., Yuba S., Saijo M., Nakatsu Y., Murai H., Nakatsuru Y., Ishikawa T., Hirota S., Kitamura Y. (1995). High incidence of ultraviolet-B-or chemical-carcinogen-induced skin tumors in mice lacking the xeroderma pigmentosum group A gene. Nature.

[79] Jhappan C., Noonan F., Merlino G. (2003). Ultraviolet radiation and cutaneous malignant melanoma. Oncogene.

[80] Botta L., Filippi S., Bizzarri B.M., Zippilli C., Meschini R., Pogni R., Baratto M.C., Villanova L., Saladino R. (2020). Synthesis and Evaluation of Artemisinin-Based Hybrid and Dimer Derivatives as Antimelanoma Agents. ACS Omega.

[81] Kotla N.G., Singh S., Maddiboyina B., Sunnapu O., Webster T.J. (2016). A novel dissolution media for testing drug release from a nanostructured polysaccharide-based colon specific drug delivery system: An approach to alternative colon media. Int. J. Nanomed..

[82] Badmus J.A., Ekpo O.E., Hussein A.A., Meyer M., Hiss D.C. (2019). Cytotoxic and cell cycle arrest properties of two steroidal alkaloids isolated from Holarrhena floribunda (G. Don) T. Durand & Schinz leaves. BMC Complement Altern. Med..

[83] Vasconcelos Z.S., Ralph A., Calcagno D.Q., Dos Santos Barbosa G., Nascimento Pedrosa T., Antony L.P., de Arruda Cardoso Smith M., de Lucas Chazin E., Vasconcelos T., Montenegro R.C. (2018). Anticancer potential of benzothiazolic derivative (E)-2-((2-(benzo[d]thiazol-2-yl)hydrazono)methyl)-4-nitrophenol against melanoma cells. Toxicol. In Vitro.

[84] Staker B.L., Feese M.D., Cushman M., Pommier Y., Zembower D., Stewart L., Burgin A.B. (2005). Structures of three classes of anticancer agents bound to the human topoisomerase I-DNA covalent complex. J. Med. Chem..

[85] Pal S., Kumar V., Kundu B., Bhattacharya D., Preethy N., Reddy M.P., Talukdar A. (2019). Ligand-based Pharmacophore Modeling, Virtual Screening and Molecular Docking Studies for Discovery of Potential Topoisomerase I Inhibitors. Comput. Struct. Biotechnol..

[86] Adasme M.F., Linnemann K.L., Bolz S.N., Kaiser F., Salentin S., Haupt V.J., Schroeder M. (2021). PLIP 2021: Expanding the scope of the protein-ligand interaction profiler to DNA and RNA. Nucleic Acids Res..

[87] Botta L., Filippi S., Zippilli C., Cesarini S., Bizzarri B.M., Cirigliano A., Rinaldi T., Paiardini A., Fiorucci D., Saladino R. (2020). Artemisinin Derivatives with Antimelanoma Activity Show Inhibitory Effect against Human DNA Topoisomerase 1. ACS Med. Chem. Lett..

[88] Mark P., Nilsson L. (2001). Structure and Dynamics of the TIP3P, SPC, and SPC/EWater Models at 298 K. J. Phys. Chem. A.

[89] Morris G.M., Huey R., Lindstrom W., Sanner M.F., Belew R.K., Goodsell D.S., Olson A.J. (2009). AutoDock4 and AutoDockTools4: Automated docking with selective receptor flexibility. J. Comput. Chem..

[90] O’Boyle N.M., Banck M., James C.A., Morley C., Vandermeersch T., Hutchison G.R. (2011). Open Babel: An open chemical toolbox. J. Cheminform..

[91] Seeliger D., de Groot B.L. (2010). Ligand docking and binding site analysis with PyMOL and Autodock/Vina. J. Comput. Aided Mol. Des..

[92] Suzuki R., Matsuno S., Sakagami H., Okada Y., Shirataki Y. (2014). Search of new cytotoxic crude materials against human oral squamous cell carcinoma using 1H NMR-based metabolomics. Anticancer Res..

