# Supplementary material for: Lignin Nanoparticles Deliver Novel Thymine Biomimetic Photo-Adducts with Antimelanoma Activity
Source: Int J Mol Sci. 2022 Jan 14;23(2):915. doi: 10.3390/ijms23020915 (PMC8777952; doi:10.3390/ijms23020915)

SUPPLEMENTARY DATA AND FIGURES

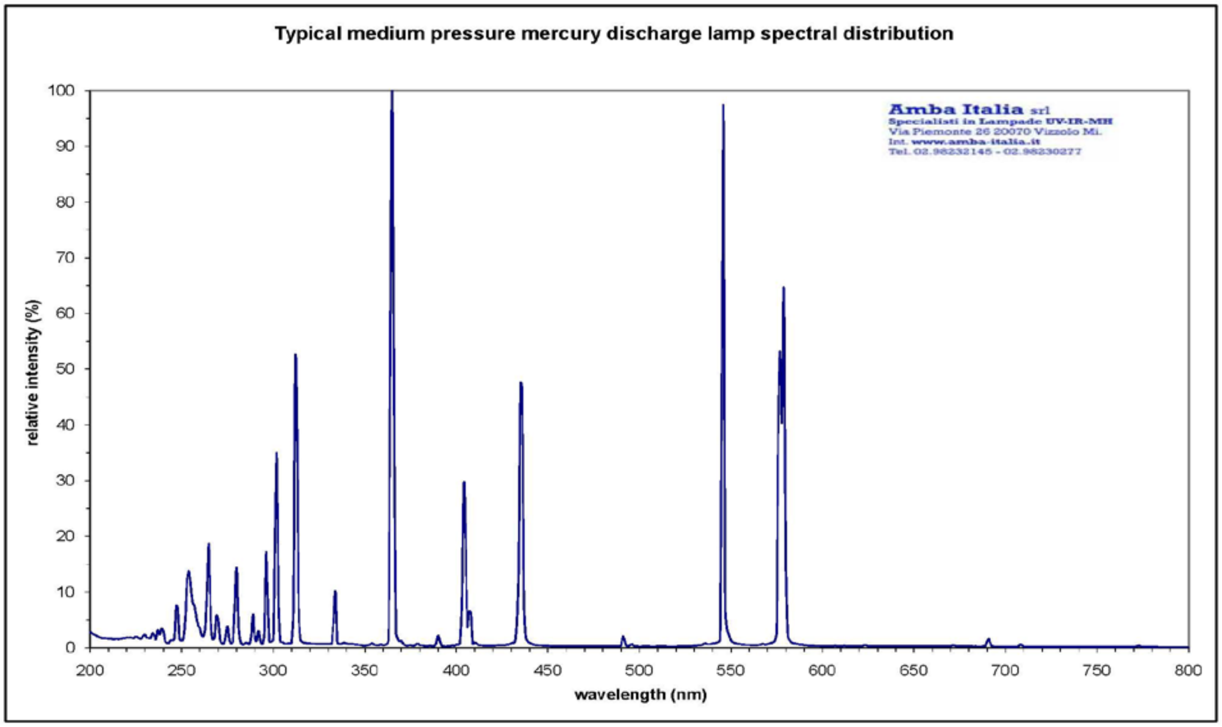

Figure S1. Haerus (250 Watt) source emission spectrum.

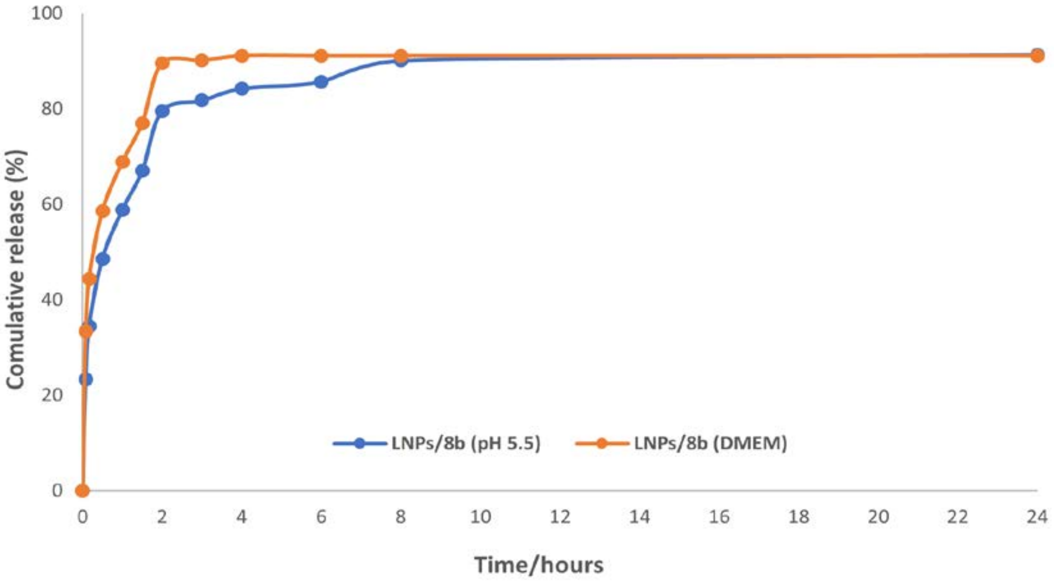

Figure S2. Kinetic release of LNPs/**8b** in the culture medium DMEM as a selected sample and compared with data referring to the buffer medium at pH 5.5.

Table S1. MTT assay of LNPs on FB789 cell line.

Type	Entry	Time (h)	FB789 (CC_{50} μM)
LNPs	1	2	12212±12.15
	2	4	12452±14.12
	3	24	12731±11.01
	4	72	14111±16.05

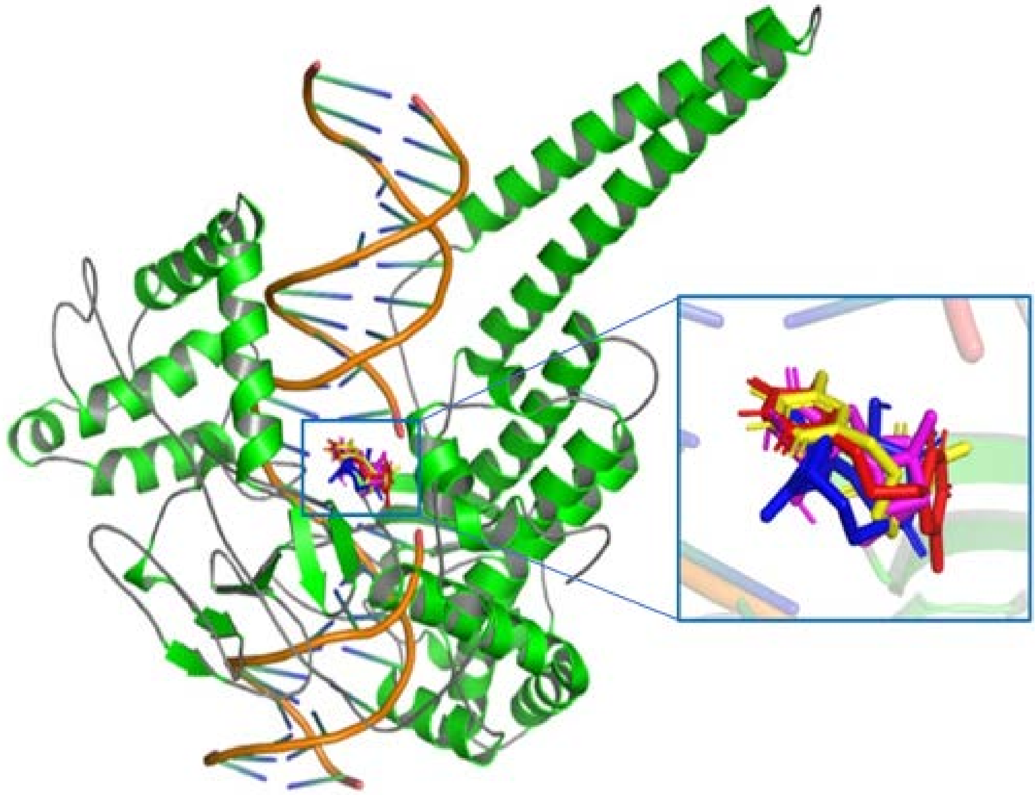

Figure S3. The conformations of bioactive compounds with the best binding affinity in complex with TOP1/DNA duplex. Binding conformations of the compounds have been provided by Autodock Vina. The following code color was applied: Compound **8a** blue; compound **8b** magenta; compound **4a** red; and compound **4b** yellow.

Figure S4. ^{1}H and ^{13}C NMR spectra of compounds **4a-d, 5a-d, 6a-d, 7a-c** and **8a-d**.

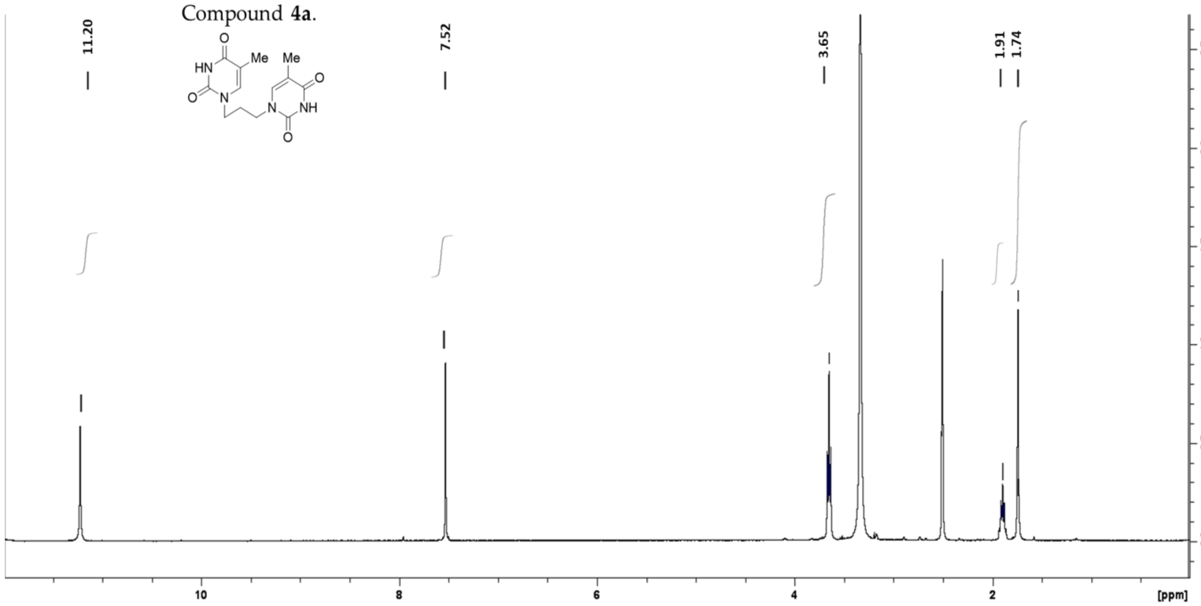

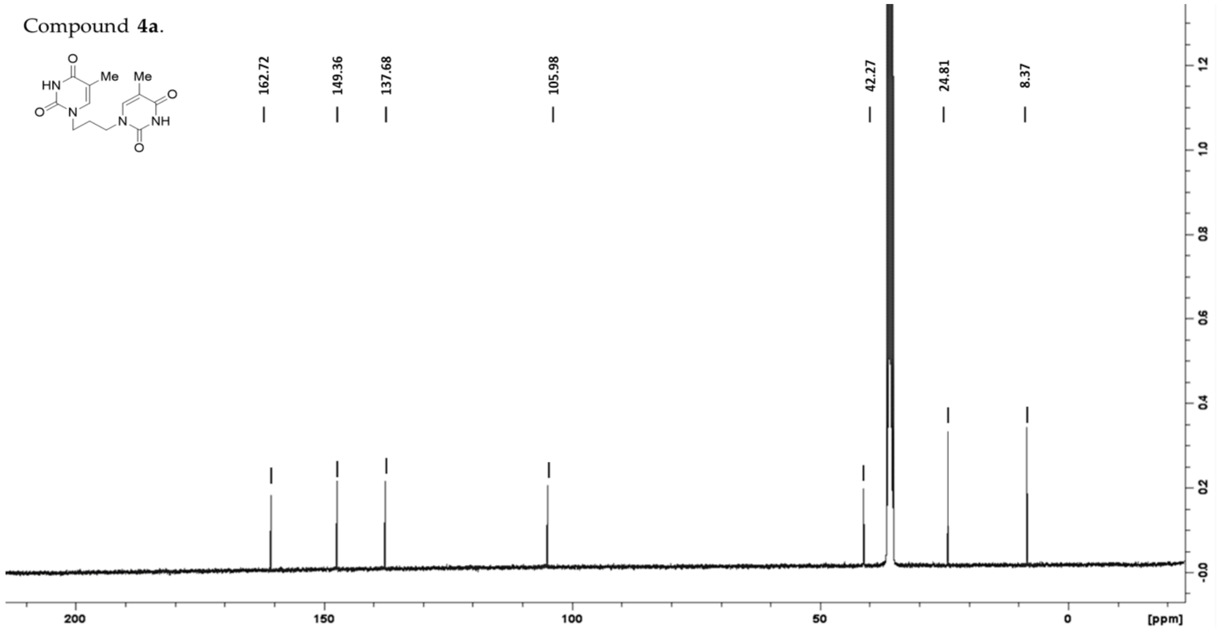

Compound **4b**.

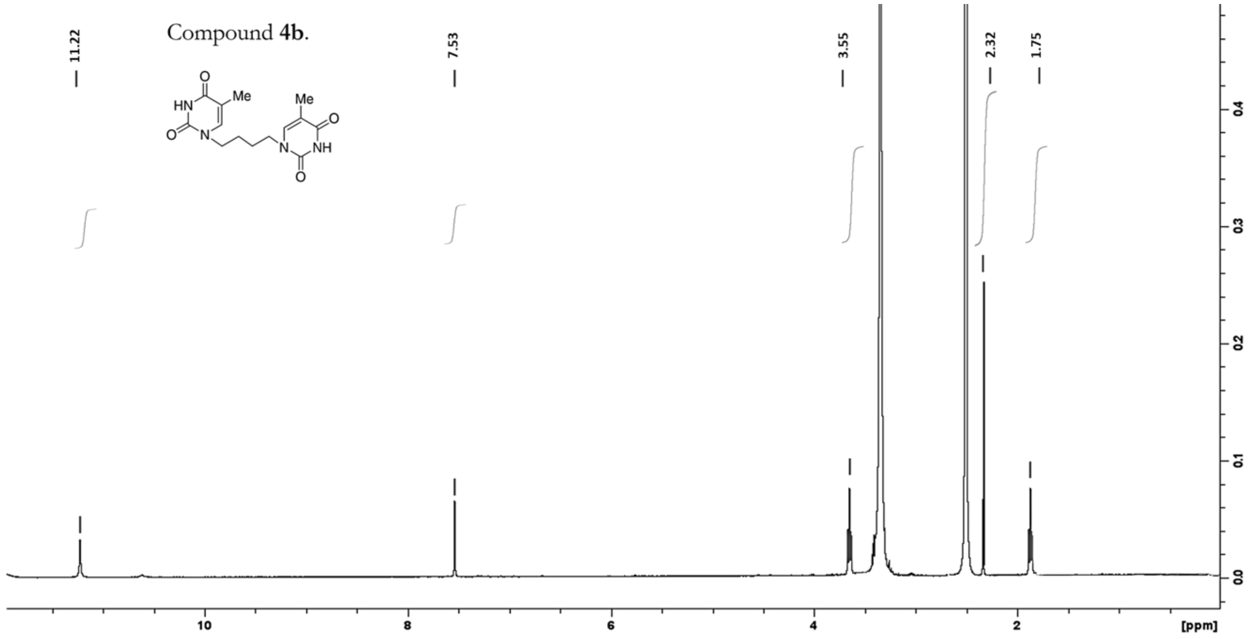

Compound **4b**.

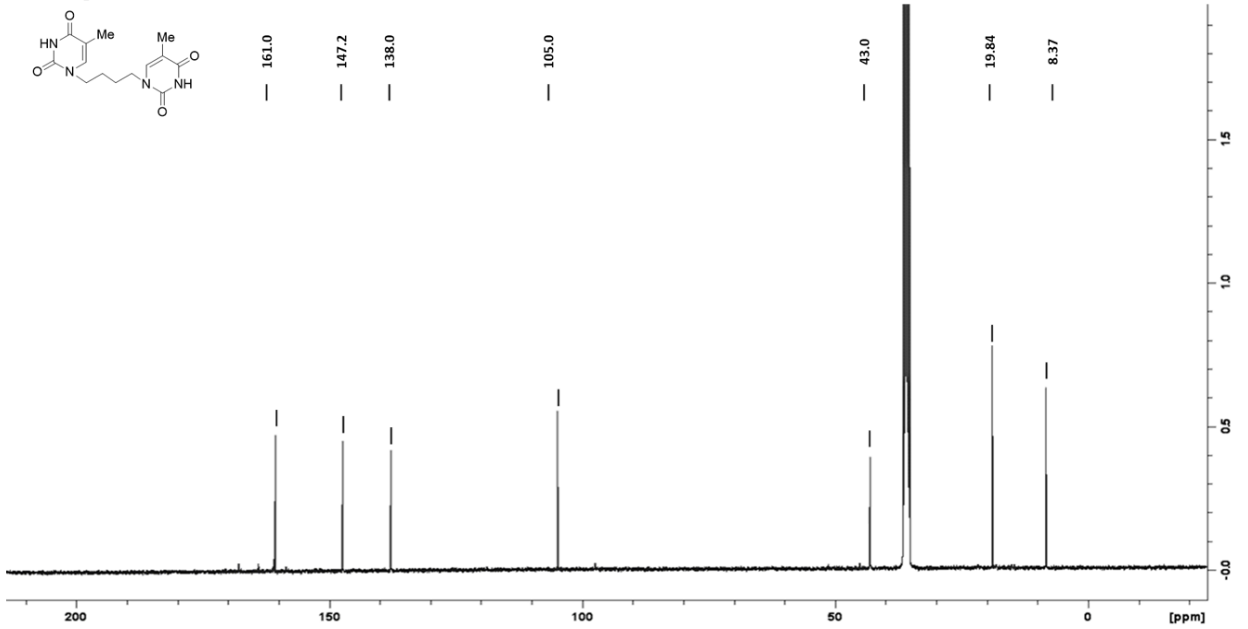

Compound **4c**.

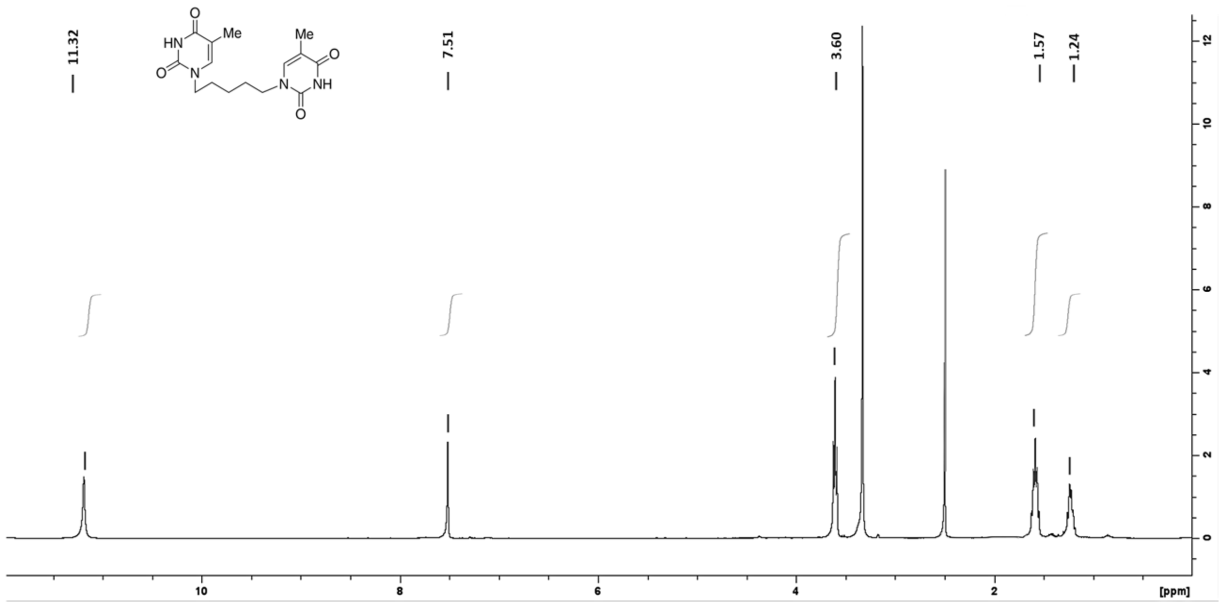

Compound **4c**.

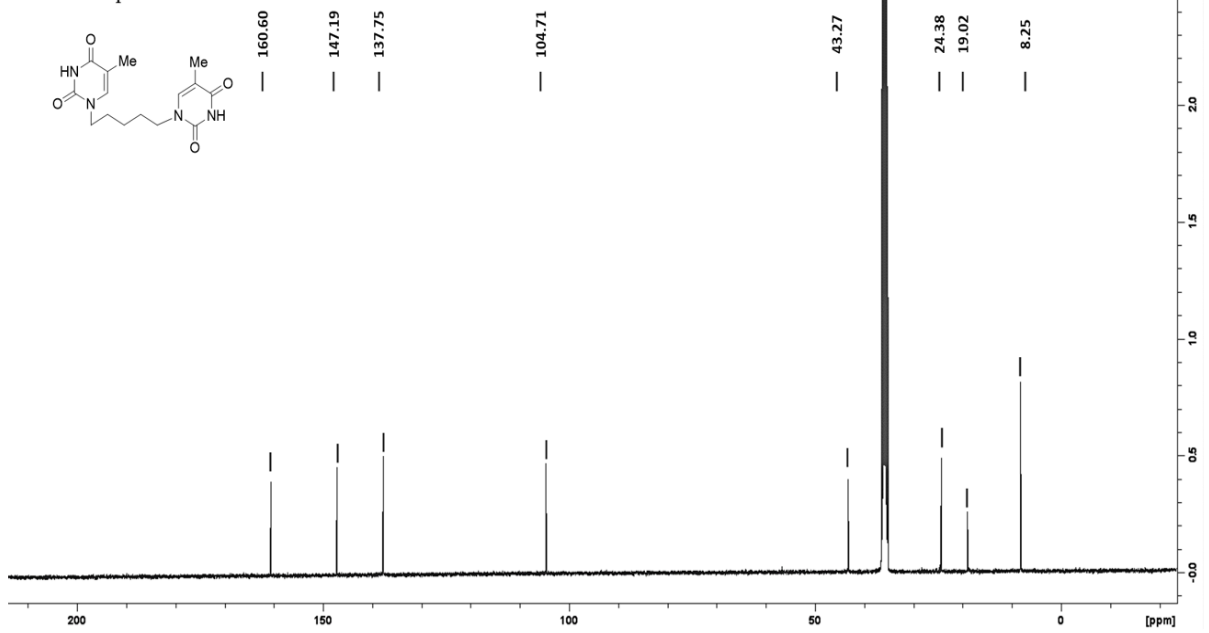

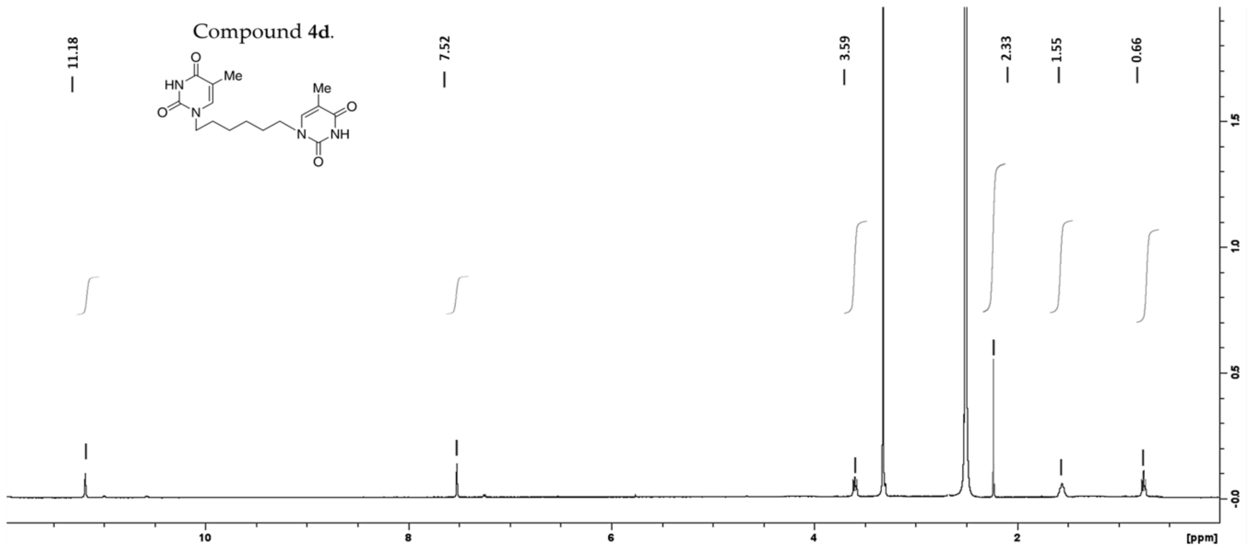
Compound 4d.
Me
HN
O
N
11.18
7.52
3.59
2.33
1.55
0.66
[ppm]

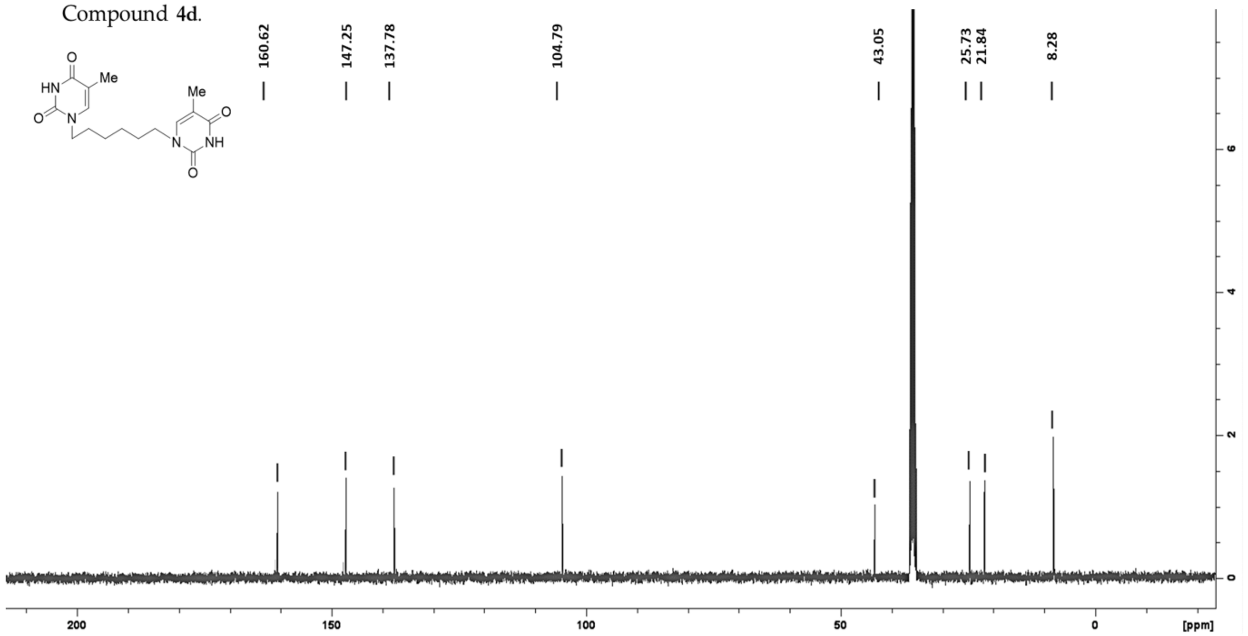
Compound 4d.
Me
HN
NH
O
N
160.62
147.25
137.78
104.79
43.05
25.73
21.84
8.28
[ppm]

Compound **5a**.

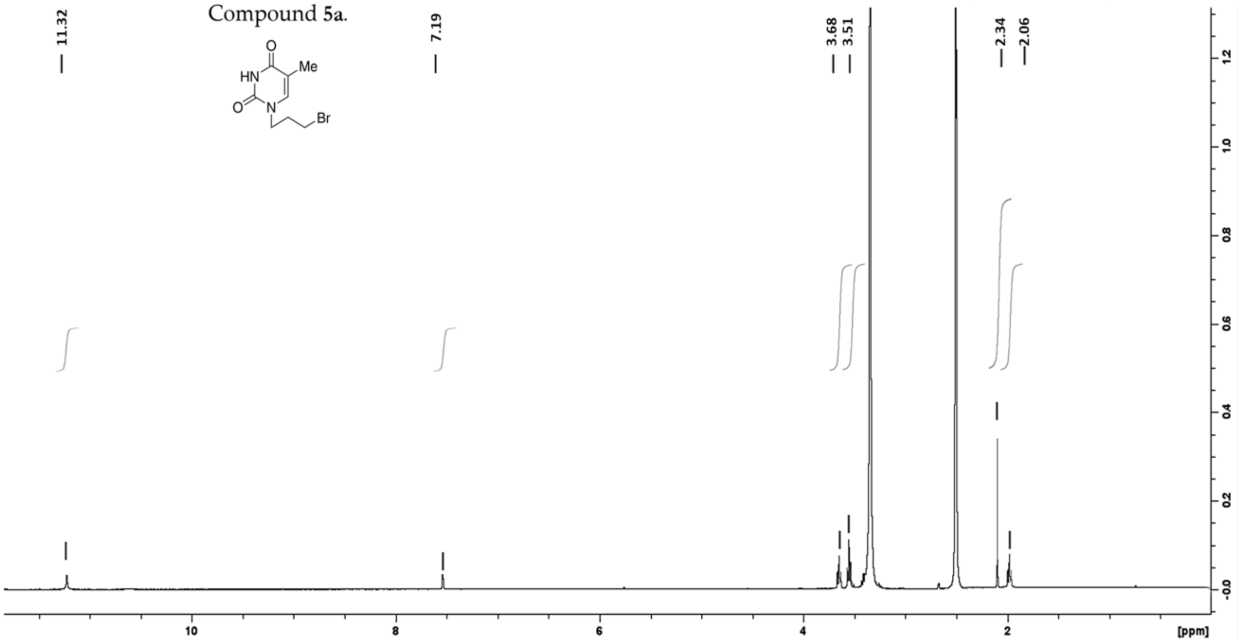

Compound **5a**.

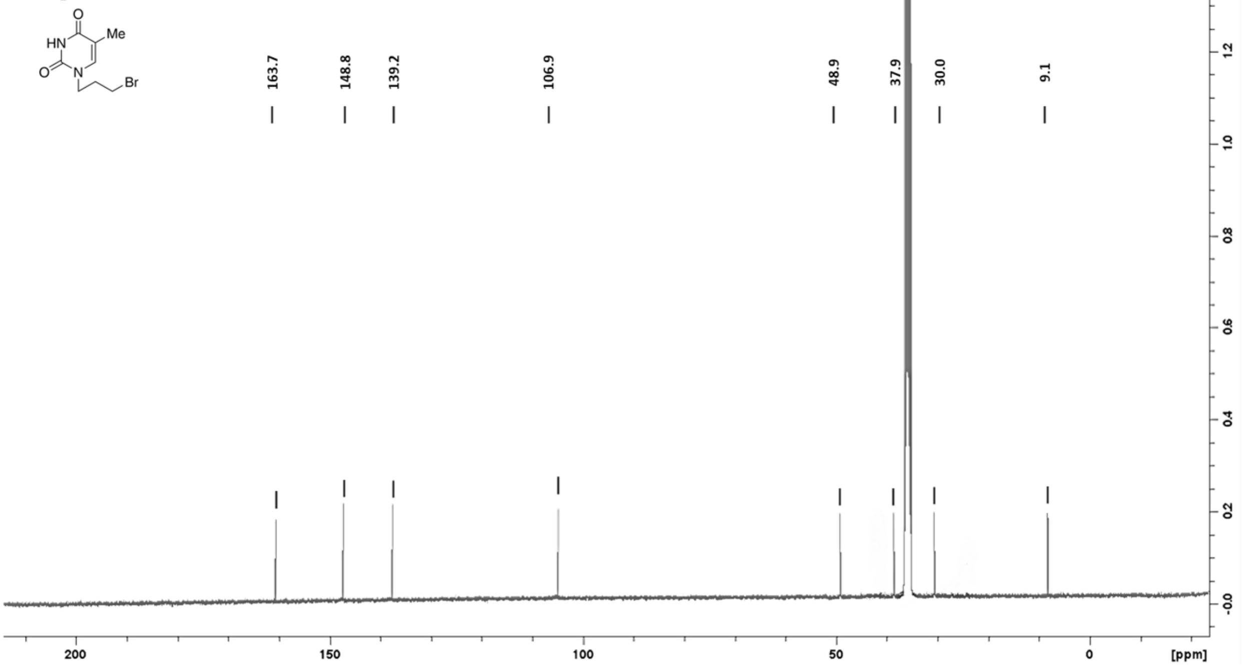

Compound **5b**.

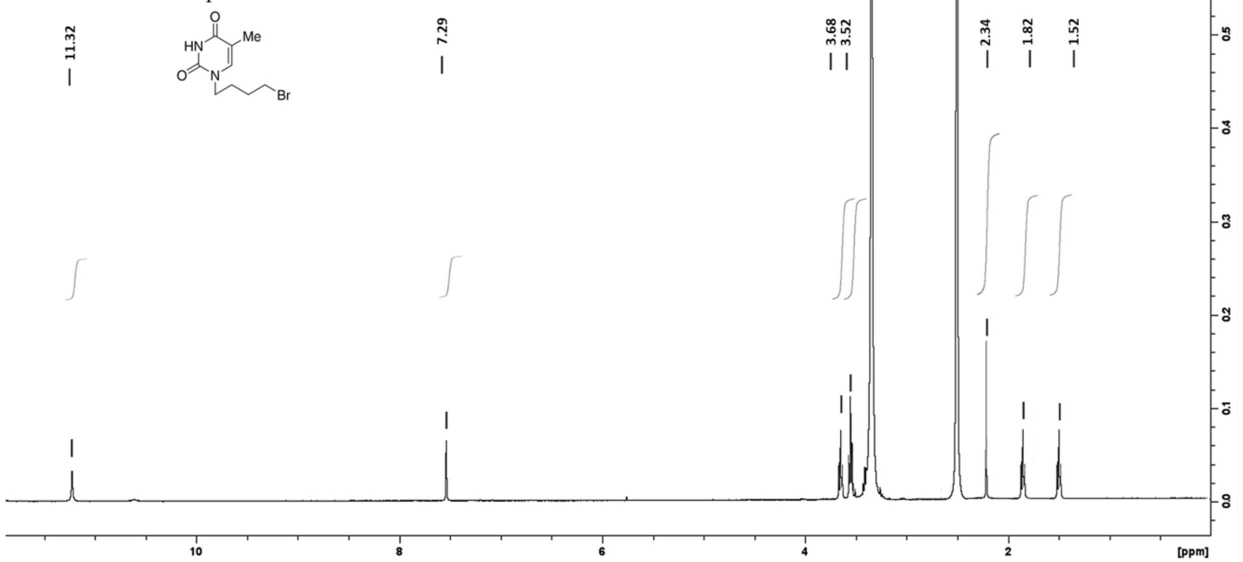

Compound **5b**.

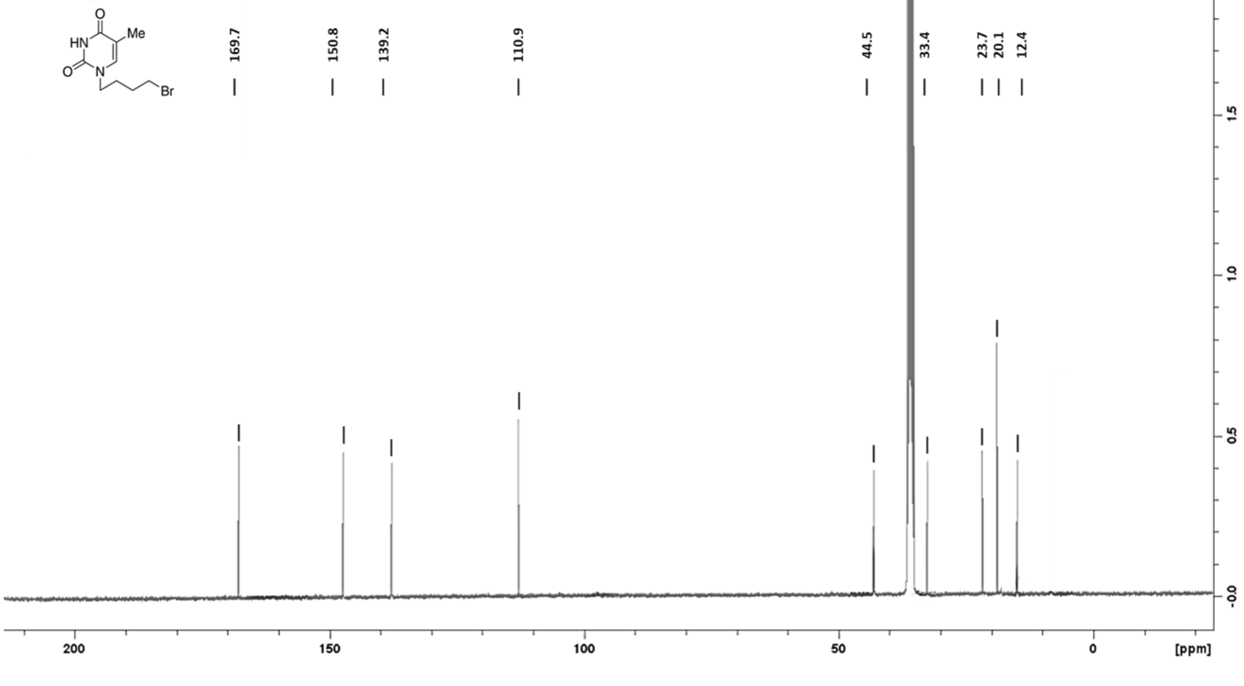

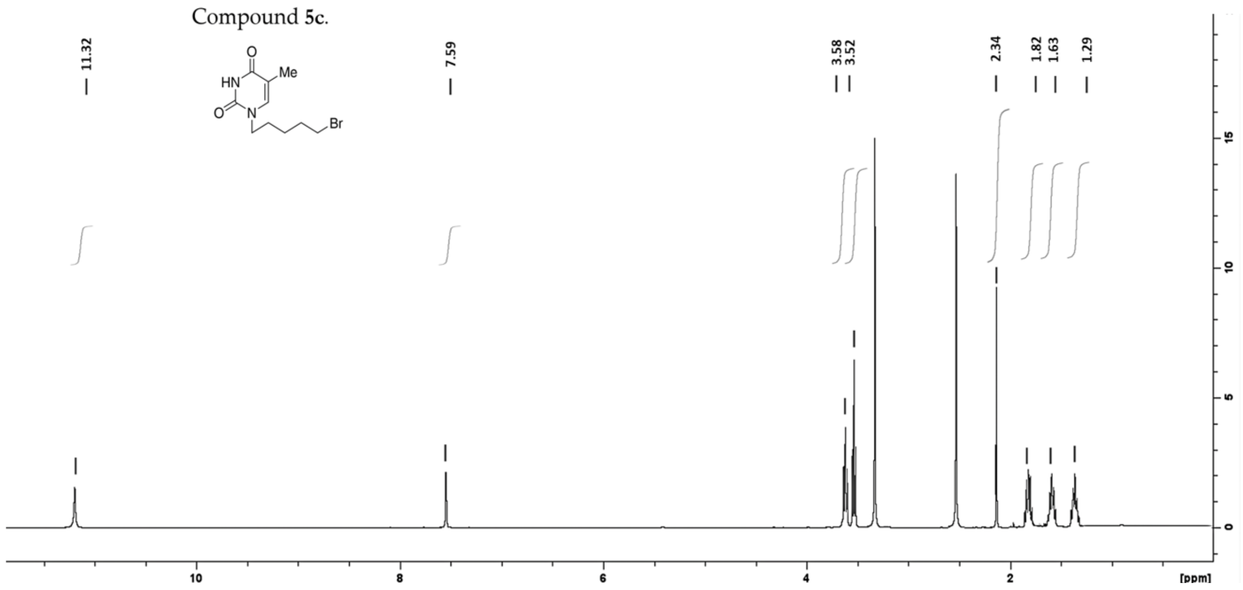
Compound 5c.
HN
O
Me
O
N
Br
11.32
7.59
3.58
3.52
2.34
1.82
1.63
1.29
10
8
6
4
2
[ppm]
15
10
5
0

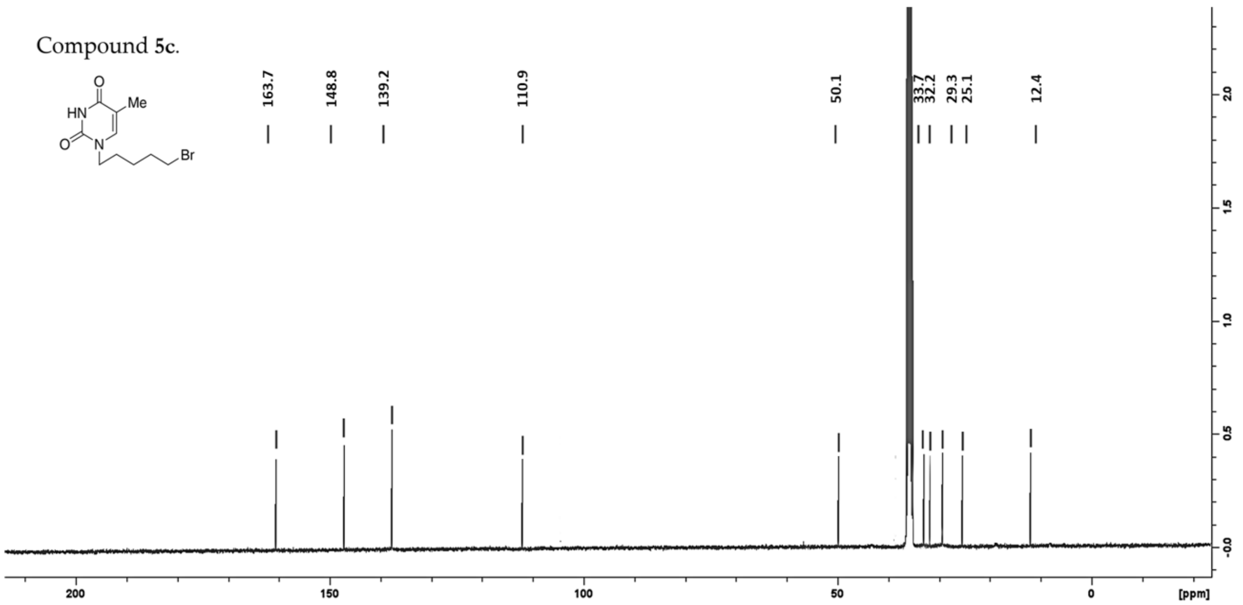
Compound 5c.
HN
O
Me
O
N
Br
163.7
148.8
139.2
110.9
50.1
33.7
32.2
29.3
25.1
12.4
200
150
100
50
0
[ppm]
2.0
1.5
1.0
0.5
0.0

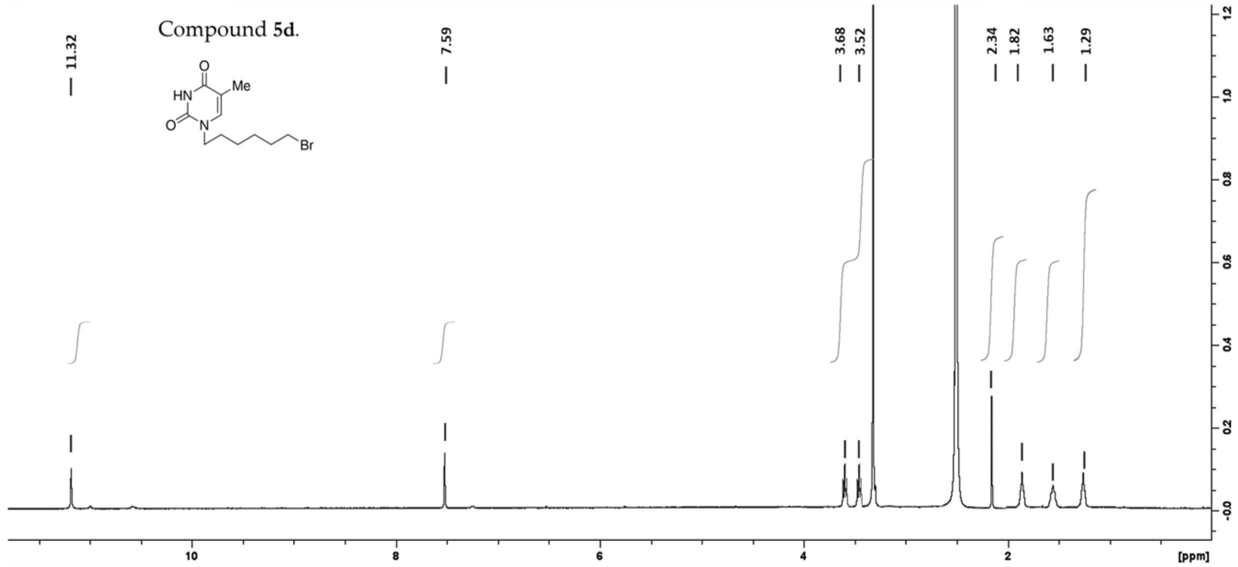
Compound 5d.
11.32
7.59
3.68
3.52
2.34
1.82
1.63
1.29
HN
O
Me
N
Br
[ppm]

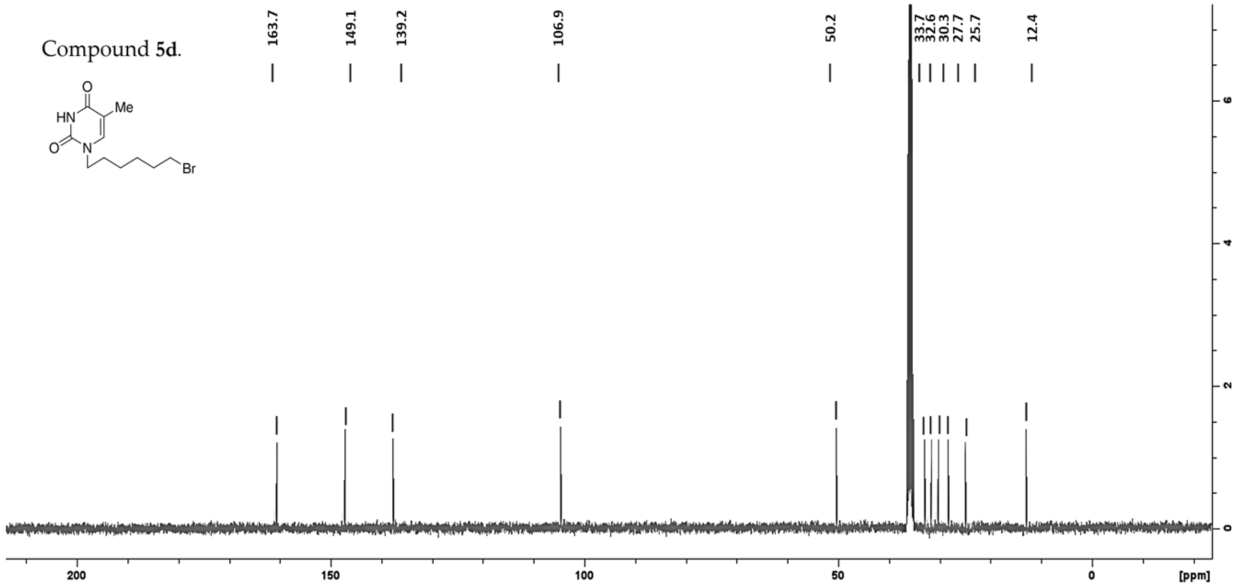
Compound 5d.
163.7
149.1
139.2
106.9
50.2
33.7
32.6
30.3
27.7
25.7
12.4
HN
O
Me
N
Br
[ppm]

Compound 6a.

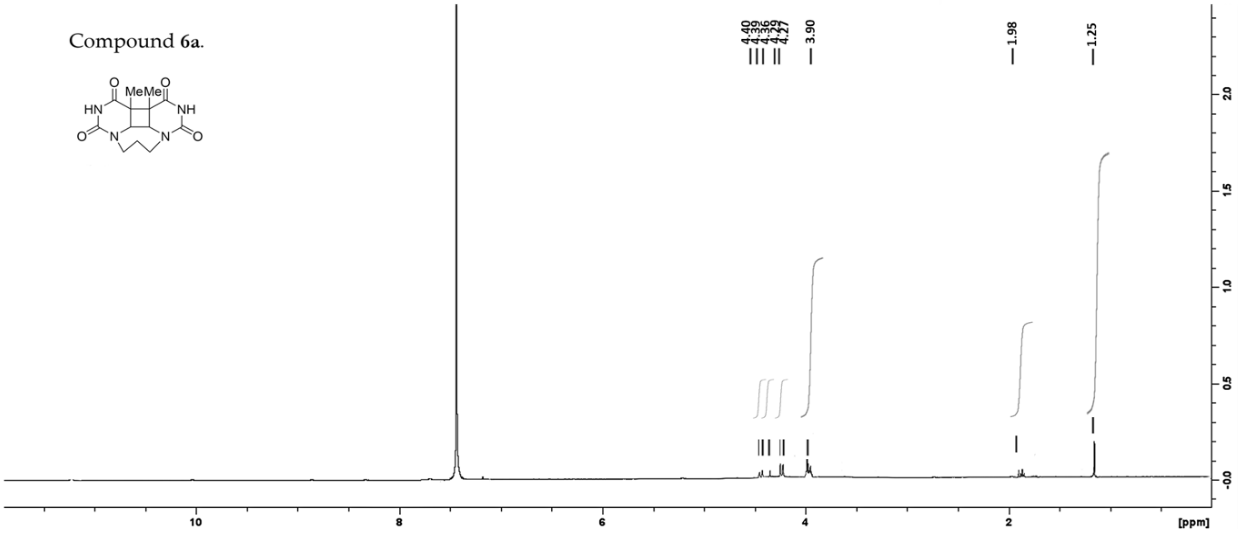

Compound 6a.

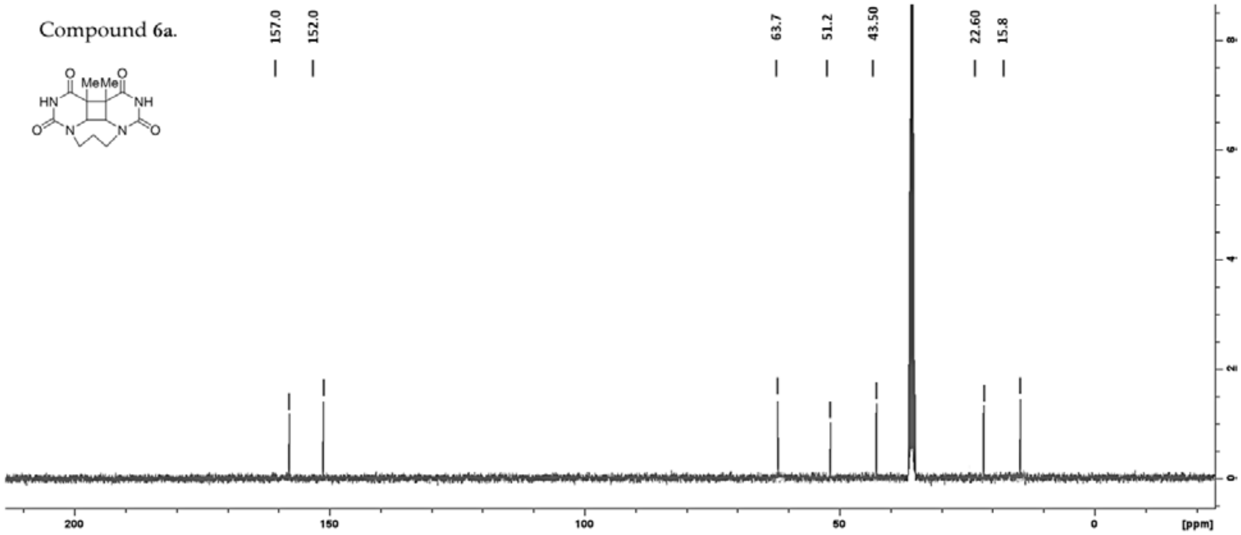

Compound **6b**.

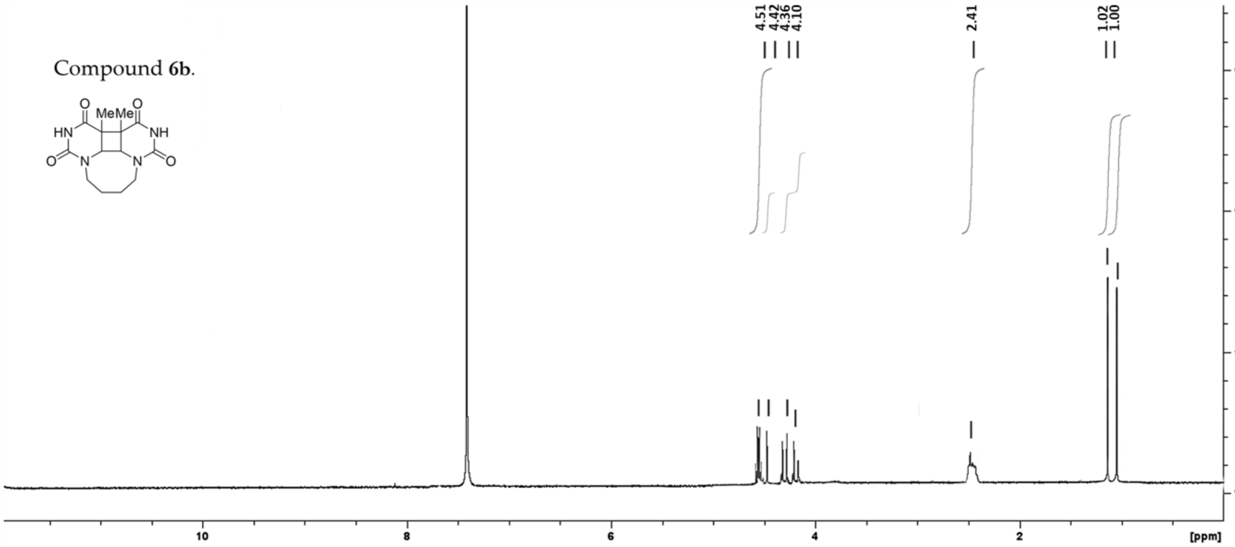

Compound **6b**.

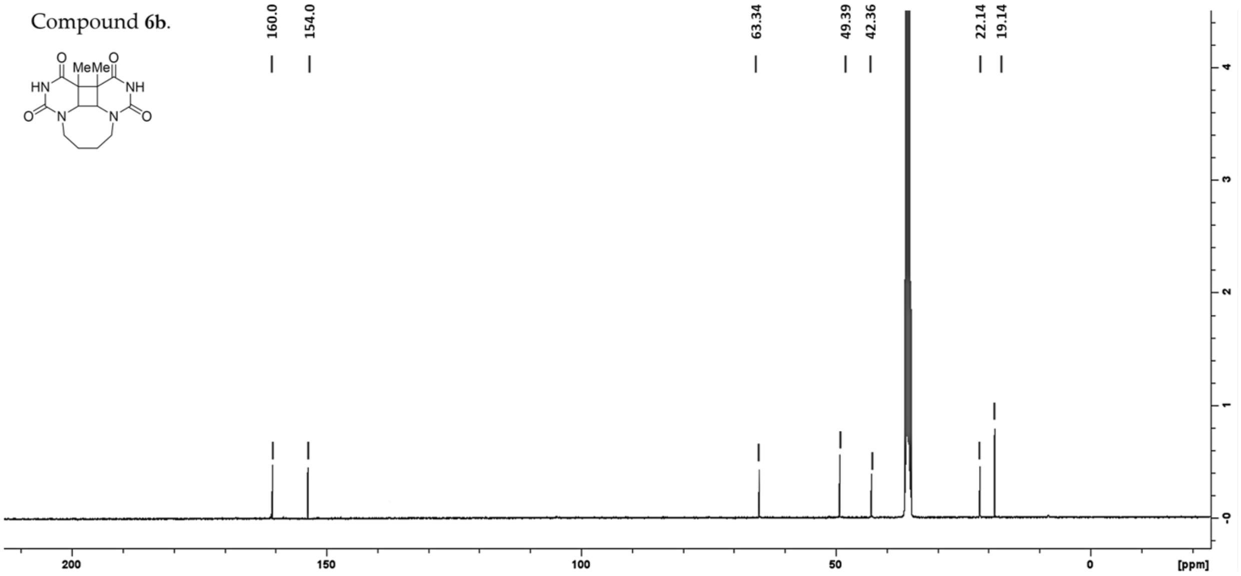

Compound 6c.

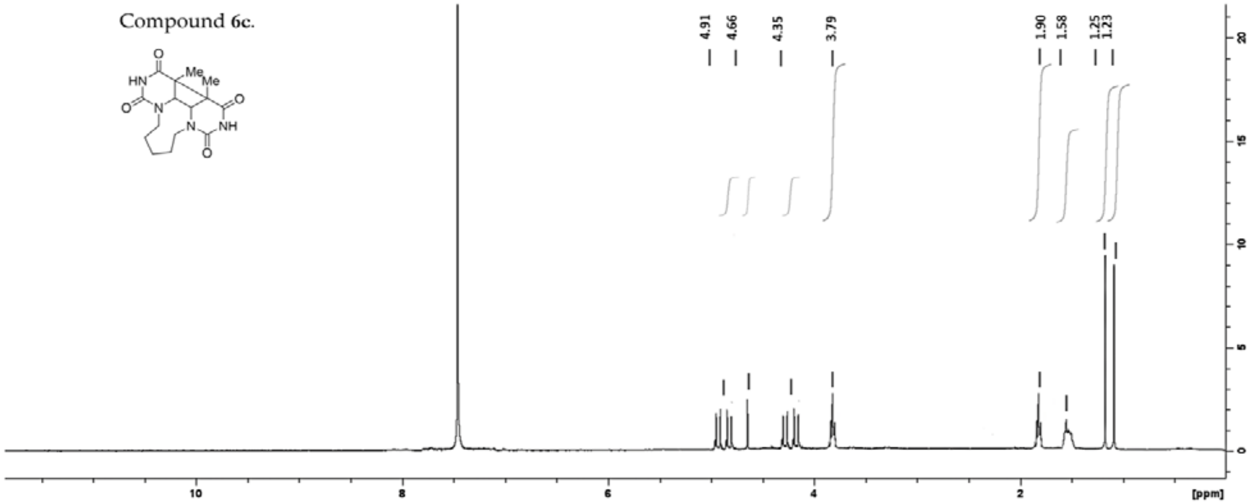

Compound 6c.

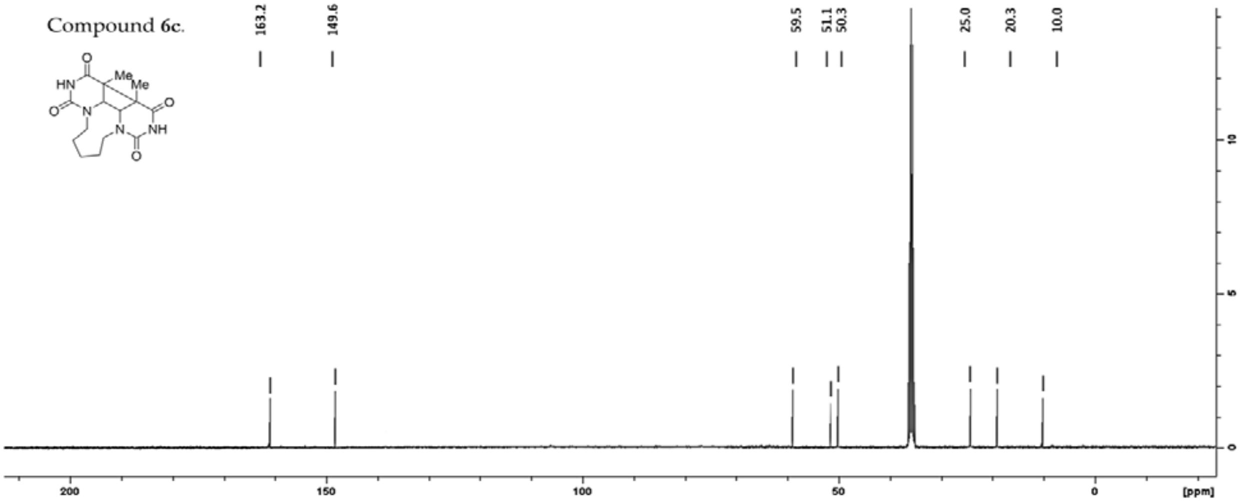

Compound **6d**.

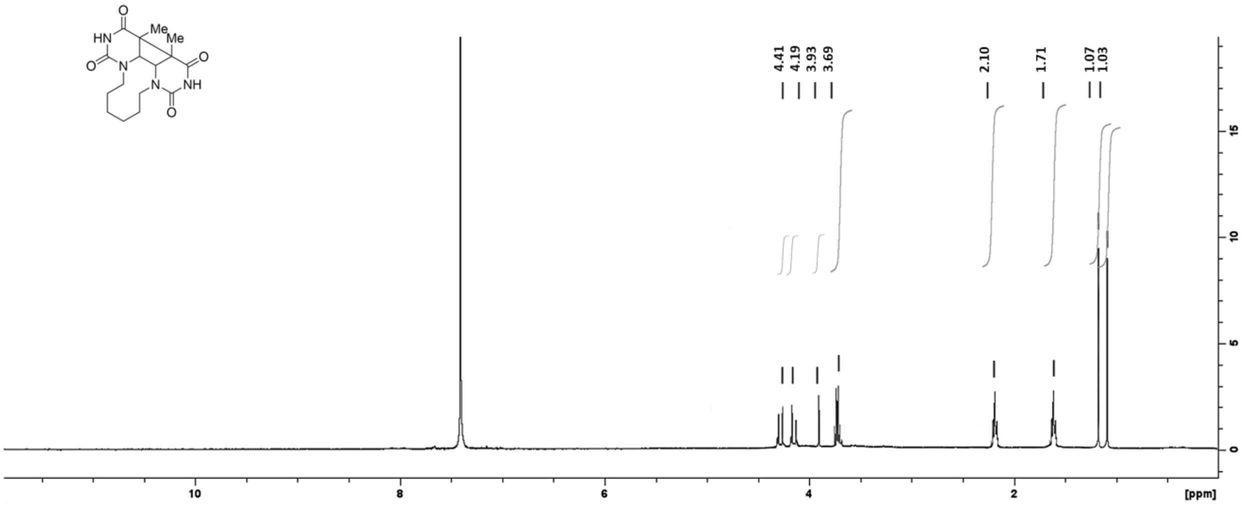

Compound **6d**.

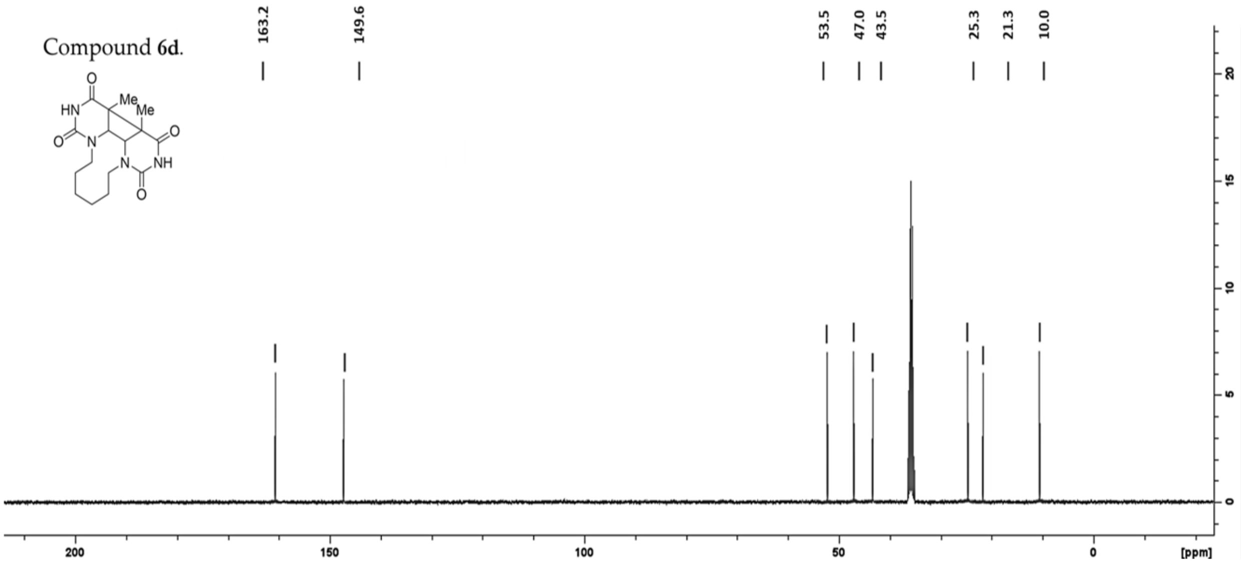

Compound 7a.

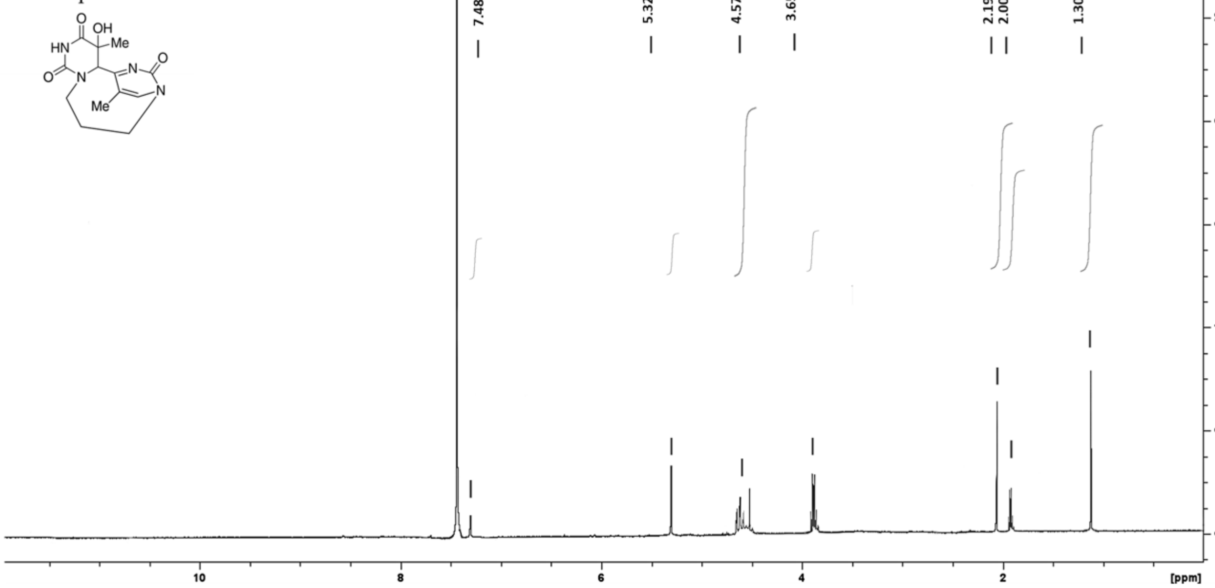

Compound 7a.

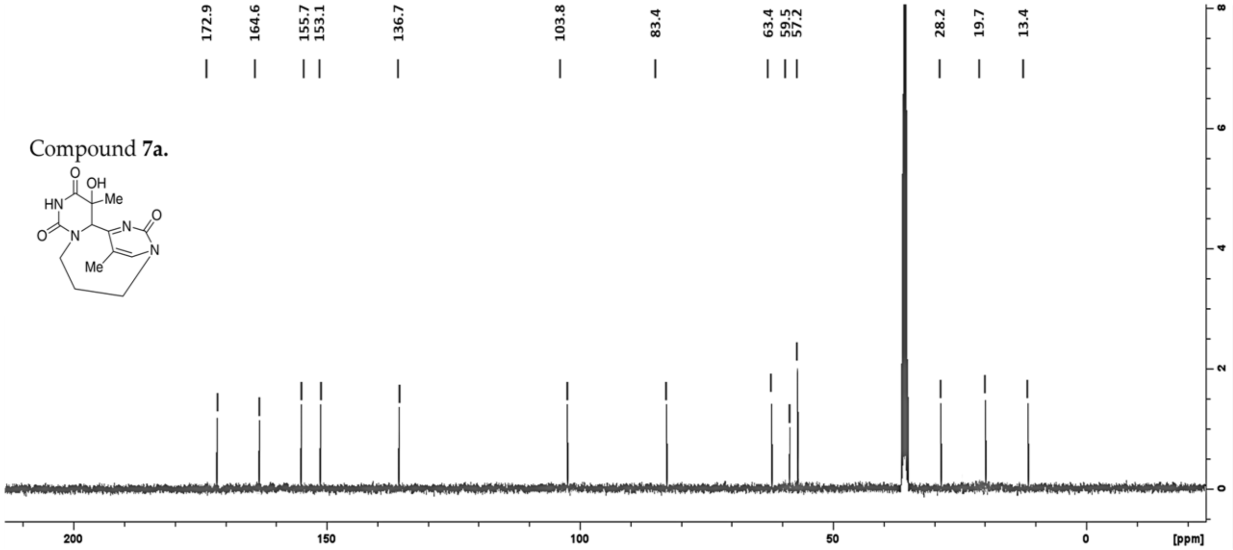

Compound **7b**.

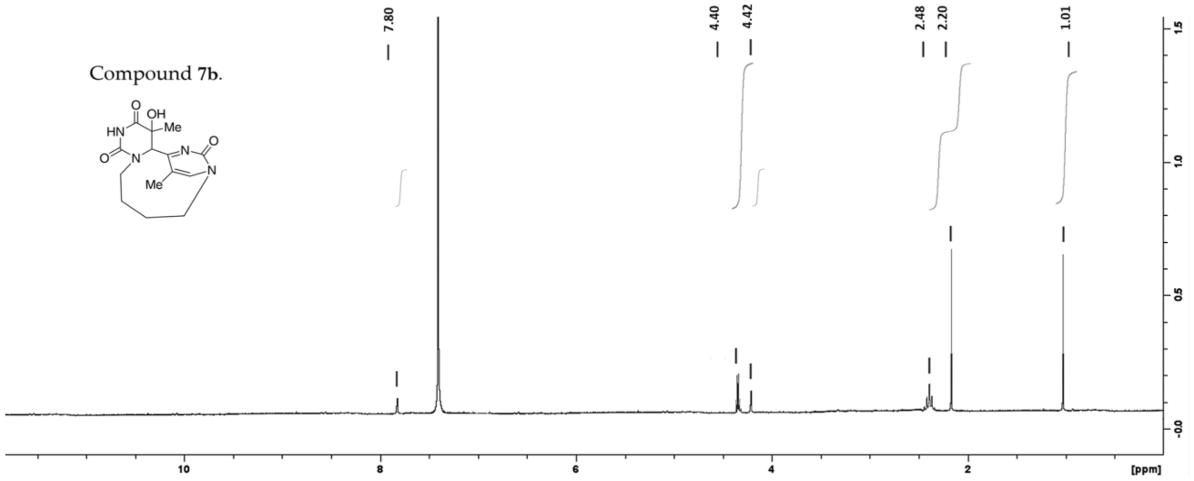

Compound **7b**.

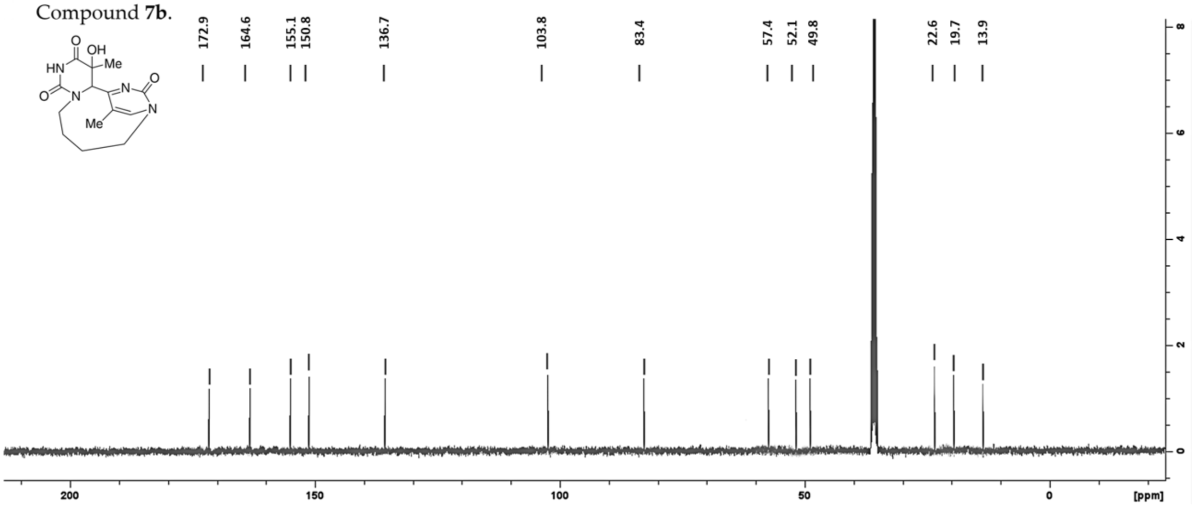

Compound **7c**.

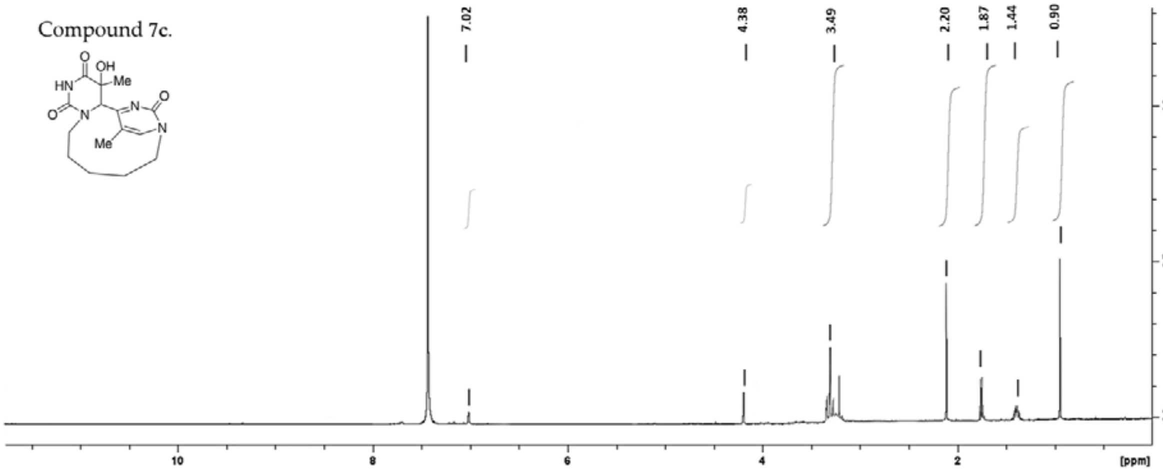

Compound **7c**.

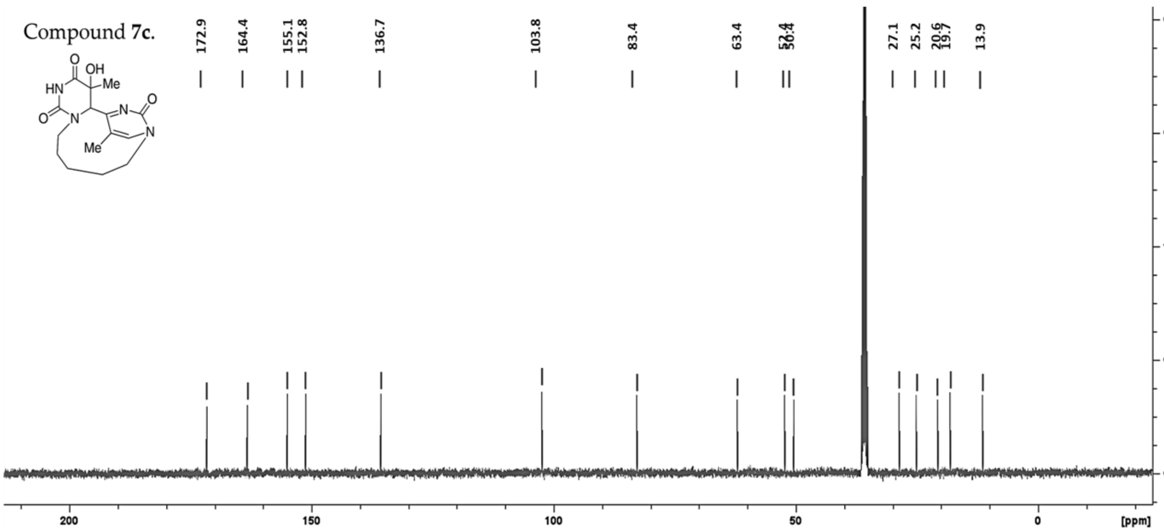

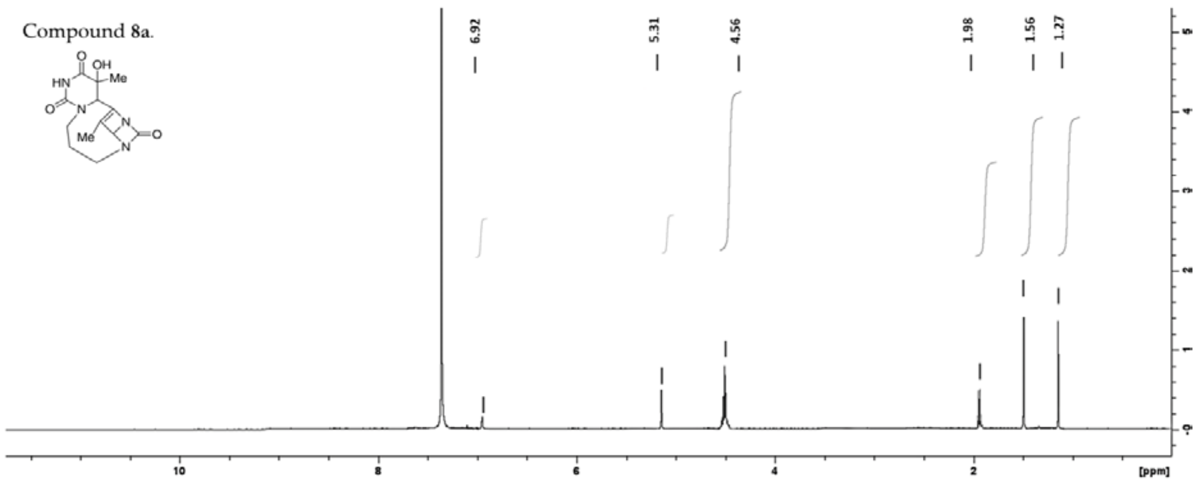
Compound 8a.
HN
O
OH
Me
N
Me
N
N
O
6.92
5.31
4.56
1.98
1.56
1.27
10
8
6
4
2
[ppm]

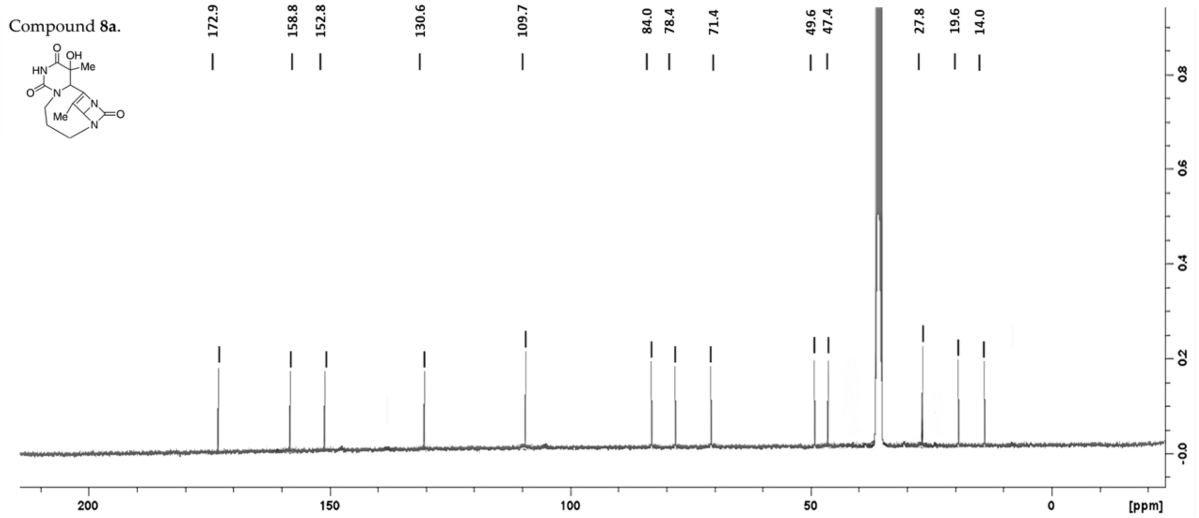
Compound 8a.
HN
O
OH
Me
N
Me
N
N
O
172.9
158.8
152.8
130.6
109.7
84.0
78.4
71.4
49.6
47.4
27.8
19.6
14.0
0.8
0.6
0.4
0.2
-0.0
200
150
100
50
0
[ppm]

Compound **8b**.

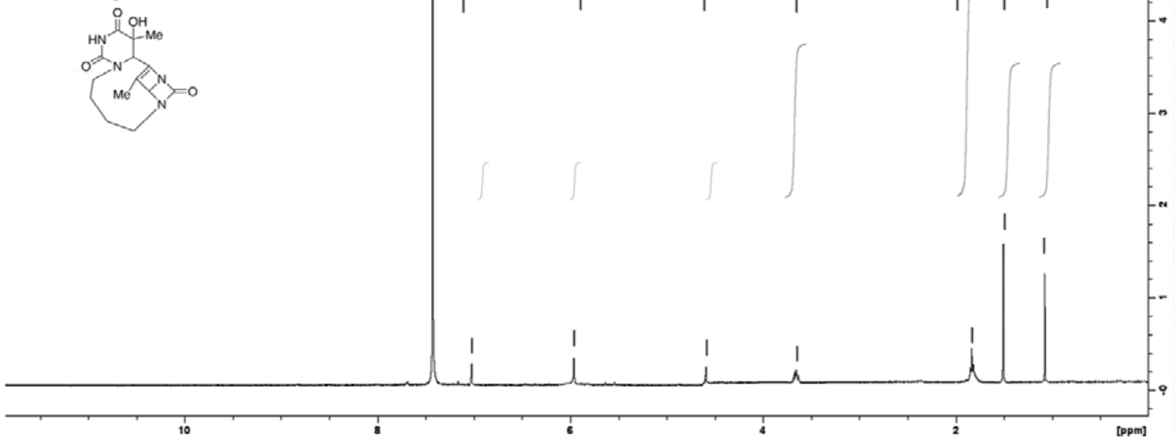

Compound **8b**.

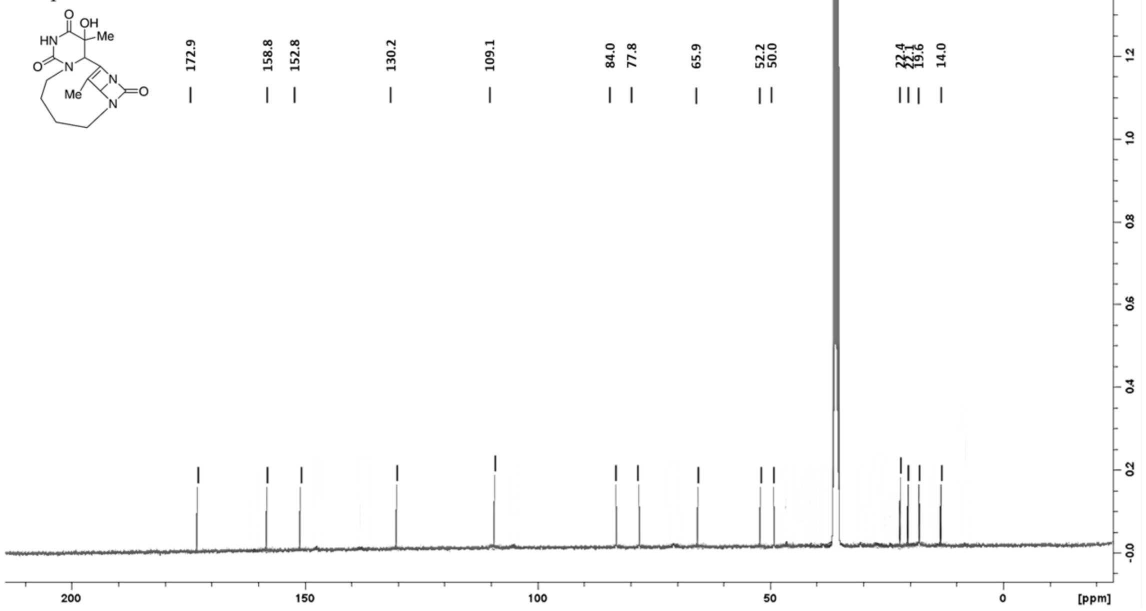

Compound **8c**.

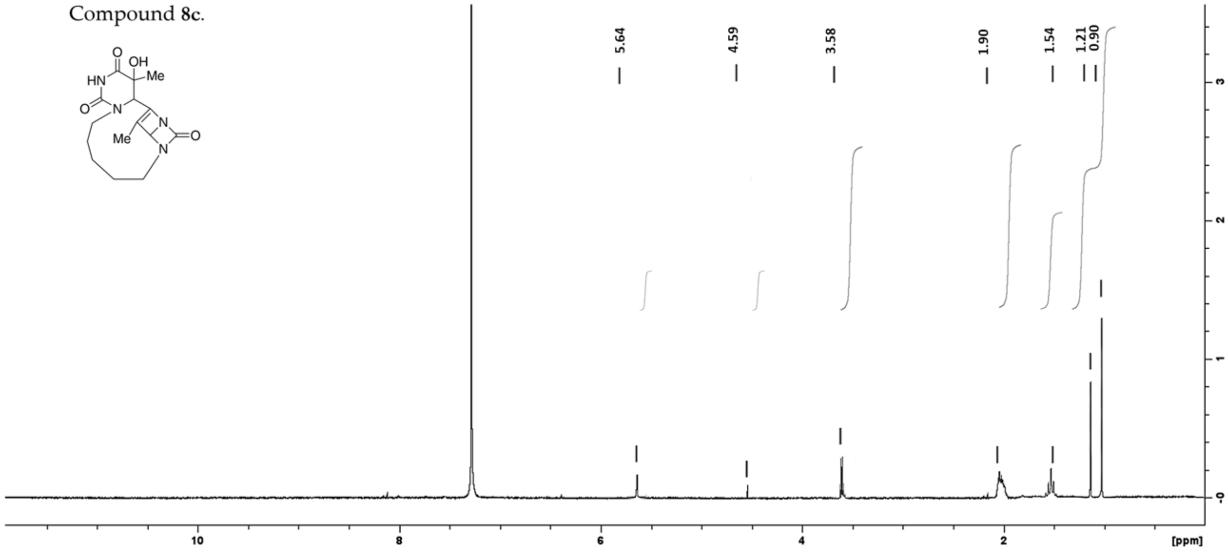

Compound **8c**.

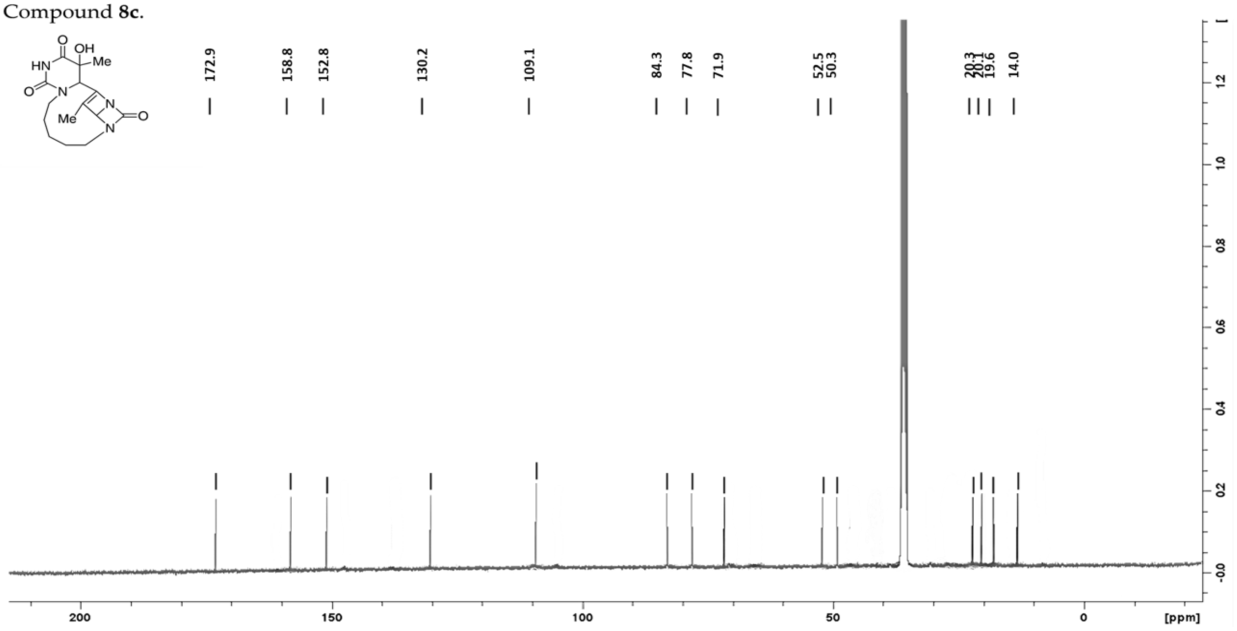

Compound **8d**.

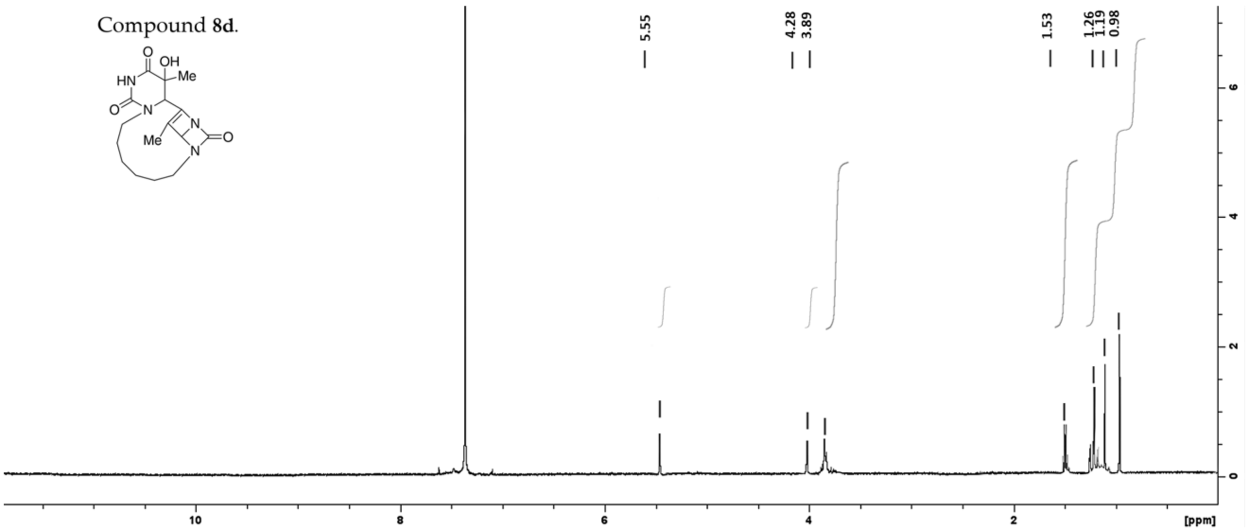

Compound **8d**.

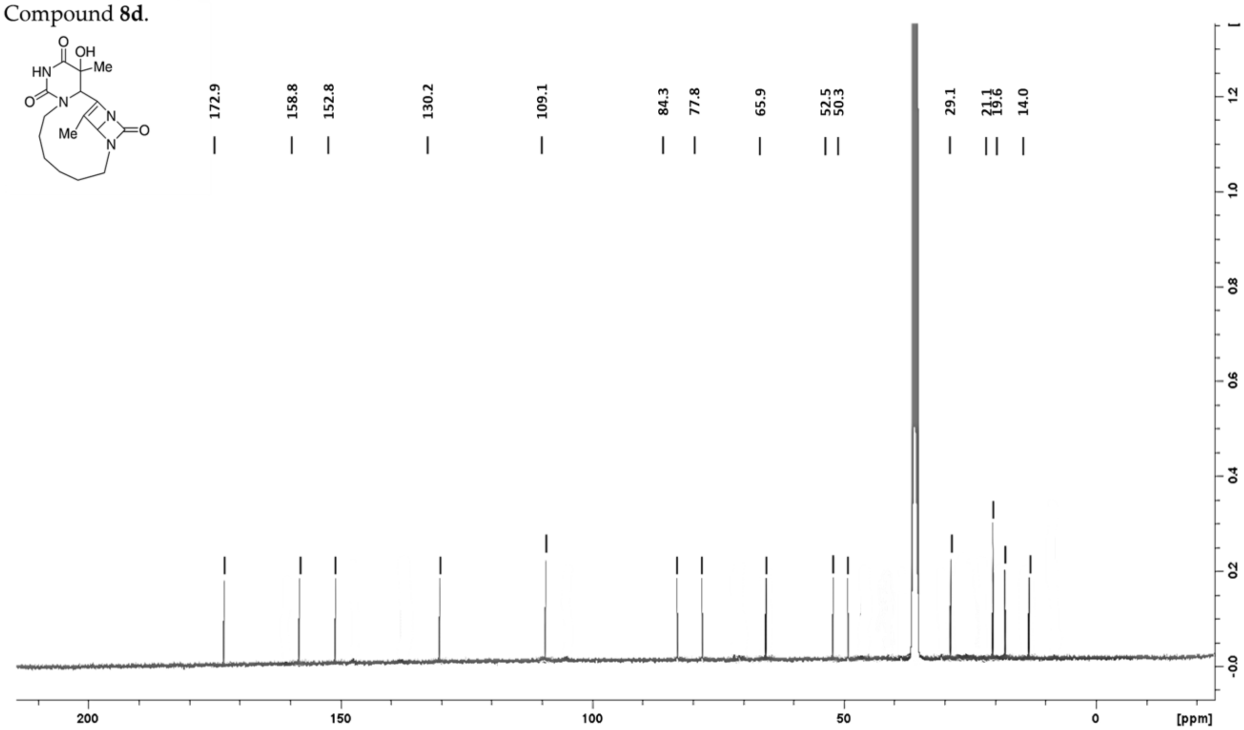

Supplement: Supplementary file 1 [file ijms-23-00915-s001.zip › ijms-1513183-supplementary.pdf]
